# Operational characteristics of full random effects modelling (‘frem’) compared to stepwise covariate modelling (‘scm’)

**DOI:** 10.1007/s10928-023-09856-w

**Published:** 2023-04-21

**Authors:** Lisa F. Amann, Sebastian G. Wicha

**Affiliations:** Department of Clinical Pharmacy, Institute of Pharmacy, University of Hamburg, Bundesstraße 45, 20146 Hamburg, Germany

**Keywords:** Covariate analysis, Population pharmacokinetics, Simulation study, NONMEM^®^

## Abstract

An adequate covariate selection is a key step in population pharmacokinetic modelling. In this study, the automated stepwise covariate modelling technique (‘scm’) was compared to full random effects modelling (‘frem’). We evaluated the power to identify a ‘true’ covariate (covariate with highest correlation to the pharmacokinetic parameter), precision, and accuracy of the parameter-covariate estimates. Furthermore, the predictive performance of the final models was assessed. The scenarios varied in covariate effect sizes, number of individuals (n = 20–500) and covariate correlations (0–90% cov-corr). The PsN ‘frem’ routine provides a 90% confidence intervals around the covariate effects. This was used to evaluate its operational characteristics for a statistical backward elimination procedure, defined as ‘frem_posthoc_’ and to facilitate the comparison to ‘scm’. ‘Frem_posthoc_’ had a higher power to detect the true covariate with lower bias in small n studies compared to ‘scm’, applied with commonly used settings (forward p < 0.05, backward p < 0.01). This finding was vice versa in a statistically similar setting. For ‘frem_posthoc_’, power, precision and accuracy of the covariate coefficient increased with higher number of individuals and covariate effect magnitudes. Without a backward elimination step ‘frem’ models provided unbiased coefficients with highly imprecise coefficients in small n datasets. Yet, precision was superior to final ‘scm’ model precision obtained using common settings. We conclude that ‘frem_posthoc_’ is also a suitable method to guide covariate selection, although intended to serve as a full model approach. However, a deliberated selection of automated methods is essential for the modeller and using those methods in small datasets needs to be taken with caution.

**Supplementary Information:**

The online version contains supplementary material available at 10.1007/s10928-023-09856-w.

## Introduction

Over the past decades, population pharmacokinetic modelling with nonlinear mixed effects (NLME) approaches efficiently supported drug development. During model development covariates are analysed to establish a relationship between a model parameter and a patient specific variable. A covariate can be any variable on patient-level (not time varying) that influences the pharmacokinetics (PK) or pharmacodynamics (PD) of a drug. If informative, it reduces unexplained inter-individual PK or PD variability. To guide dose adjustments in special patient populations (e.g. elderly, adipose, hepatically or renally impaired patients), a covariate analysis is also of interest to regulatory authorities [1]. To date, a number of automated covariate selection techniques are available [2]: these include e.g. stepwise covariate modelling (‘scm’) [3], or least absolute shrinkage and selection operator (lasso) [4]. The stepwise procedure tests predefined covariates on structural PK or PD parameters of interest. Automated covariate selection methods are statistically driven methods. The ‘scm’ includes covariates by the highest drop of objective function (dOFV) with a predefined p-value during the forward inclusion. In one of the more common implementations covariates are included until the likelihood ratio test identifies no significant covariate parameter relationship anymore. Afterwards, the backward elimination reduces the covariate model to obtain the final model, by applying a stricter p-value. This method has been evaluated on their properties and compared to other established methods before [5, 6]. In contrast to that, the ‘frem’ is a full model approach and includes all covariates of interest as observations (i.e., explicitly defining the likelihood of the covariate values) [7]. A full covariance matrix quantifies the random effects of PK parameters and describes parameter covariate relationships [8]. With the matrix, covariances of covariates can inform for other covariates so that this method is less sensitive to collinearity. Covariate coefficients are obtained from the ratio of covariance between parameter and covariate variability to the covariate variance [7].

The novel ‘frem’ method has not been applied to many clinical datasets yet [9–11]. Although ‘scm’ and ‘frem’ are techniques that are rather complementary in nature due their inherently different way to approach covariate modelling, a structured comparison of the operational characteristics using a simulation study is lacking. The aim of this study was to compare the ‘scm’ and ‘frem’ as automated covariate analysis methods. In order to enable a comparison, we here introduce the ‘frem_posthoc_’ that offers a covariate selection step from the final ‘frem’ model using the confidence intervals around the estimated covariate effect sizes in the final ‘frem’ model. In the present study, the following aspects between ‘scm’, ‘frem’ and ‘frem_posthoc_’ were compared: (1) the power to identify the true covariate (here defined as the covariate with the highest correlation with the PK parameter), (2) accuracy and precision of the estimated relationship, as well as (3) the predictive performance. To enable a thorough comparison, we investigated the impact of dataset size (n = 20–500), and covariate correlation (0–90%) for three covariate effect sizes in sparse simulated datasets using the commonly used (‘scm’) or predefined (‘frem’/’frem_posthoc_’) settings of both approaches as well as statistically equal settings.

## Methods

**Fig. 1:**
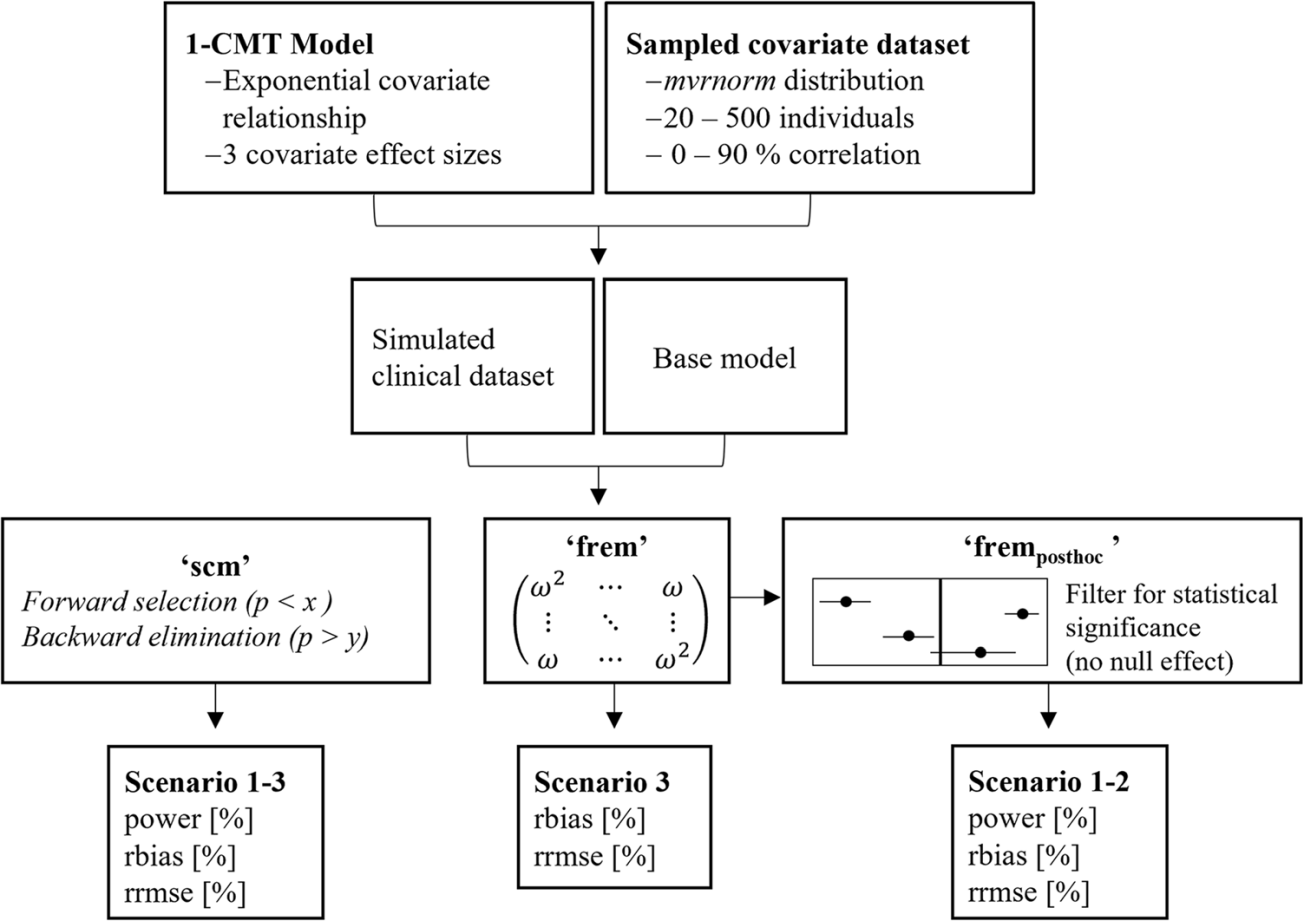
Graphical workflow of the simulation study. *Scm* stepwise covariate modelling, *frem* full random effects modelling, *rrmse* relative root mean squared error, *rbias* relative bias, *mvrnorm* multivariate normal distribution

The workflow of this simulation study is shown in Fig. 1. The simulation dataset contained three covariates sampled from a multivariate normal distribution. The dataset was used to simulate with a one compartment model including the true covariate relationship on clearance. These simulated clinical datasets served for ‘scm’ and ‘frem’ analyses (n = 1000 for each scenario). Based upon the final models, power, precision, and accuracy were evaluated. The following section describes the single steps in detail.

### Software

NLME modelling was applied with NONMEM^®^ 7.5.0 [12], controlled through PsN 5.0.0 [13]. The software R (version 3.6.0) [14] was used for automated run executions and data analysis. The NONMEM^®^ models as well as relevant R code are provided in Supplement 1.

### Generation of datasets and simulation of PK data

#### Continuous covariates

Three vectors of three covariates (i.e., covariate_I,_ covariate_II_ and covariate_III_) with defined means, and variances were drawn from a multivariate normal distribution (Supplement 3, Figure S3-1). The datasets included various correlations of covariate_I_ (cov_true_) and covariate_II_ from 0 to 90%. Covariate_III_ represented pure “noise” and was independent from cov_true_ and covariate_II_. All simulations used individually simulated datasets with 20, 50, 100 or 500 virtual patients (n) including 2 (sparse) concentration-time points per individual. The sparse sampling datasets included samples in the sixth and twelfth dosing interval (1 and 11.5 h time after last dose, respectively). PK profiles of the scenarios (1-CMT PK model, i.v. short infusion, linear elimination) were obtained via Monte Carlo simulations. The true PK model (run001) is described in Supplement 1.1. The simulated dose was 100 mg q12 h with 30 min infusion. The PK model parameters were clearance (CL) of 18 L/h with inter-individual variability on CL (IIV_CL_: 0.1 variance, log-normal distribution), central volume of distribution (V1) of 400 L and a residual proportional error (%CV) of 15%. Cov_true_ was implemented as an exponential covariate on CL ($\theta_{CL }$) with the $\theta_{cov }$ as covariate coefficient (Eq. 1):

1 $$CL = \theta_{CL } \, \cdot \,e^{\left( {\theta_{cov } \, \cdot \,\left( {COV - COV_{mean} } \right)} \right)} \, \cdot \,e^{\eta_i } .$$

The individual covariate value (cov) was normalized by the mean of the covariate distribution $\left( {cov - cov_{mean} } \right)$. The remaining unexplained inter-individual variability ($\eta_i$) described the individual deviation from the typical parameter $\theta_{CL{ }} { }$ for the ith individual (Eq. 1).

The observed concentration $Y_{observed,i,j}$ was calculated by the predicted concentration $Y_{predicted,i,j}$ multiplied by the proportional residual unexplained variance per individual *i* at each time point *j* (Eq. 2). No inter-occasion variability was included:

2 $$Y_{observed,i,j} = Y_{predicted, i,j} \, \cdot \,\left( {1 + \varepsilon_{prop,i,j} } \right).$$

The simulated covariate effect magnitudes varied between $\theta_{cov}$ = 0.026, 0.032 and 0.045, respectively. This resulted in relative effect sizes of  − 18 to + 22%, − 22 to + 27% and − 29 to + 41% on CL at the 5th − 95th percentile of covariate values.

### Evaluation using ‘scm’ or ‘frem’ models

Parameter estimation was performed using first order conditional estimation with interaction (FOCE+I), allowing three minimum retries on each simulated dataset (for each scenario, n = 1000). The structural model used for estimation is described in Supplement 1.2 (run002). The ADVAN 1 subroutine was used as analytical solution of the 1-CMT model. All three previously simulated covariates in the simulated dataset were provided to the ‘scm’, as well to ‘frem’ for analysis. The ‘scm’ and ‘frem’ were executed on each simulated dataset. The final ‘scm’ model results were either obtained in the last forward/backward step, or if the covariate identification failed, no covariate model was obtained. The ‘frem’ is a full model approach that includes all provided covariates simultaneously. Thereby, results cannot be compared to ‘scm’ without restrictions. To address the fundamental differences of these methods we evaluated the results in three settings:

(i) Scenario 1 evaluated the operational characteristics of ‘frem_posthoc_’. A covariate backward elimination from final ‘frem’ models was performed via the 90% confidence intervals of the estimated covariate effect and compared to final ‘scm’ models obtained with commonly used settings (forward inclusion, p < 0.05 and a backward elimination p < 0.01).
(ii) Scenario 2 assessed a statistical ‘head-to-head’ comparison of ‘frem_posthoc_’ and ‘scm’ cov_true_ coefficients with only forward inclusion (p < 0.1)
(iii) Scenario 3 showed a comparison of all estimated ‘frem’ cov_true_ covariate coefficients without a selection step compared to ‘scm’ results of Scenario 1.

#### Scenario 1

A forward selection with a p-value of < 0.05 and a backward elimination (p < 0.01) was used reflecting the commonly used settings of the ‘scm’. We compared those ‘scm’ runs, which selected cov_true_ to those ‘frem’ runs that identified cov_true_ with a covariate effect significantly different from zero. The significance was interpreted by the 90% confidence interval obtained from sampling importance resampling (SIR) [15]. The results were extracted from the PsN provided results files (PsN 5.0.0), and the effect sizes (5th – 95th percentile of the covariate effect, 90% confidence interval) reflect the default setting of the ‘frem’ PsN routine. Since this setting evaluated a backward elimination, we define this use case of the ‘frem’ as ‘frem_posthoc_’. We furthermore defined power (1–type II error) as the frequency of selecting cov_true_ in the covariate model (‘scm’), or as frequency to identify cov_true_ as a covariate with the highest effect size different from zero and non-overlapping 90% confidence interval (‘frem_posthoc_’). For the ‘frem_posthoc_’, the estimated univariate θ_cov_ coefficient was evaluated (PsN ‘frem_results.csv’), which represents the effect of a single covariate in isolation [7]. Conditional accuracy and conditional precision, expressed as rbias (Eq. 3) and rrmse (Eq. 4), were calculated as follows for significant cov_true_ coefficients:

3 $$rBIAS (\% ) = \frac{1}{N}\, \cdot \,\mathop \sum \limits_1^i \frac{(estimated_i - true_i )}{{true_i }}\, \cdot \,100,$$

4 $$rRMSE(\% ) = \sqrt {\frac{1}{N}\, \cdot \,\mathop \sum \limits_1^i \frac{(estimated_i - true_i )^2 }{{true_i^2 }} } \, \cdot \,100.$$

The denominator (N) was different across the simulated scenarios and methods, as the number of simulations for which cov_true_ coefficients was evaluated changed accordingly.

Moreover, true alpha values (Type-I error rate) were evaluated based on cov_III_ inclusion in the forward ‘scm’ models and the final ‘frem_posthoc_’ models. Cov_III_ is independent of the others and represents pure noise without having any simulated relationship between the pharmacokinetics and cov_III_. The alpha values in the final ‘frem_posthoc_’ models were defined as the frequency of runs in which the cov_III_ effect was not overlapping with zero.

According to Ribbing et al., we calculated the fraction of predictive models by assuming an estimated covariate coefficient between zero and two times cov_true_ to be likely to improve the predictive performance of a model [16]. For each scenario the fraction of predictive models was calculated (Eq. 5), where *e* represents the covariate effect size, *c* the correlation between cov_true_ and cov_II_ and *N* the dataset size varying from n = 20–500. S_ecnN_ represented the models which included cov_true_ (‘scm’) for the respective scenario. For ‘frem_posthoc_’ coefficients, s_ecnN_ represented all runs or those including a significant cov_true_ relationship with the highest effect of all three covariates in the models for comparison to the ‘scm’.

Fraction of predictive model:

5 $$s_{ecN} = 100 \, \cdot \, \frac{{\sum_{n = 1}^{1000} \left( {P_{ecnN} \, \cdot \, s_{ecnN} } \right)}}{{\sum_{n = 1}^{1000} s_{ecnN} }}(\% ),$$

where,

$$P_{ecnN} = \left\{ {\begin{array}{*{20}l} 1 \hfill & {\quad if \left| {\frac{{\hat{\theta }_{ecnN} - \theta_{ecnN} }}{{\theta_{ecnN} }}} \right| < 1} \hfill \\ 0 \hfill & {\quad otherwise} \hfill \\ \end{array} } \right..$$

#### Scenario 2

For a comparison of equal selection criteria, ‘scm’ runs with only forward inclusion (p-value < 0.1) were compared to ‘frem_posthoc_’ results (which evaluates overlap/non-overlap with zero of the 90% confidence interval). Settings for ‘frem_posthoc_’ were not changed compared to scenario 1. Power, conditional accuracy and precision were calculated for those runs, where the included cov_true_ was statistically significant. Similar to scenario 1, the predictive performance of final ‘scm’ and frem_posthoc_’ models was evaluated according to Ribbing et al. [16]. As the number of significant runs changed across the simulated scenarios (e.g. n, covariate effect magnitude, cov-corr) the denominator to calculate these evaluation metrices changed between also between both methods.

#### Scenario 3

In this scenario, conditional accuracy, and precision, but also the predictive performance of all estimated ‘frem’ cov_true_ coefficients (i.e. no posthoc selection step from the final ‘frem’ model) were compared to ‘scm’ models obtained in scenario 1.

#### Categorical covariates

Additionally, a simulation study (n = 500) with a true dichotomous categorical covariate was performed. The dataset size varied from n = 20–500 and covariate correlation to a continuous covariate was 0% or 80%. The third covariate (continuous) was independent of the others. The true model included the categorical covariate as a fractional change of clearance with an effect size of either  − 20% or  − 40%. IIV_CL_, but also inter individual variability on central volume of distribution (IIVV_c_) was included in the model. More details on this study are described in Supplement 2.

## Results

### Power of cov_true_ inclusion for ‘scm’ and ‘frem_posthoc_’

The power to include the cov_true_ throughout the investigated scenarios was highly variable. Overall, the power to select cov_true_ increased with dataset size or covariate effect and decreased in presence of covariate collinearity.

**Fig. 2:**
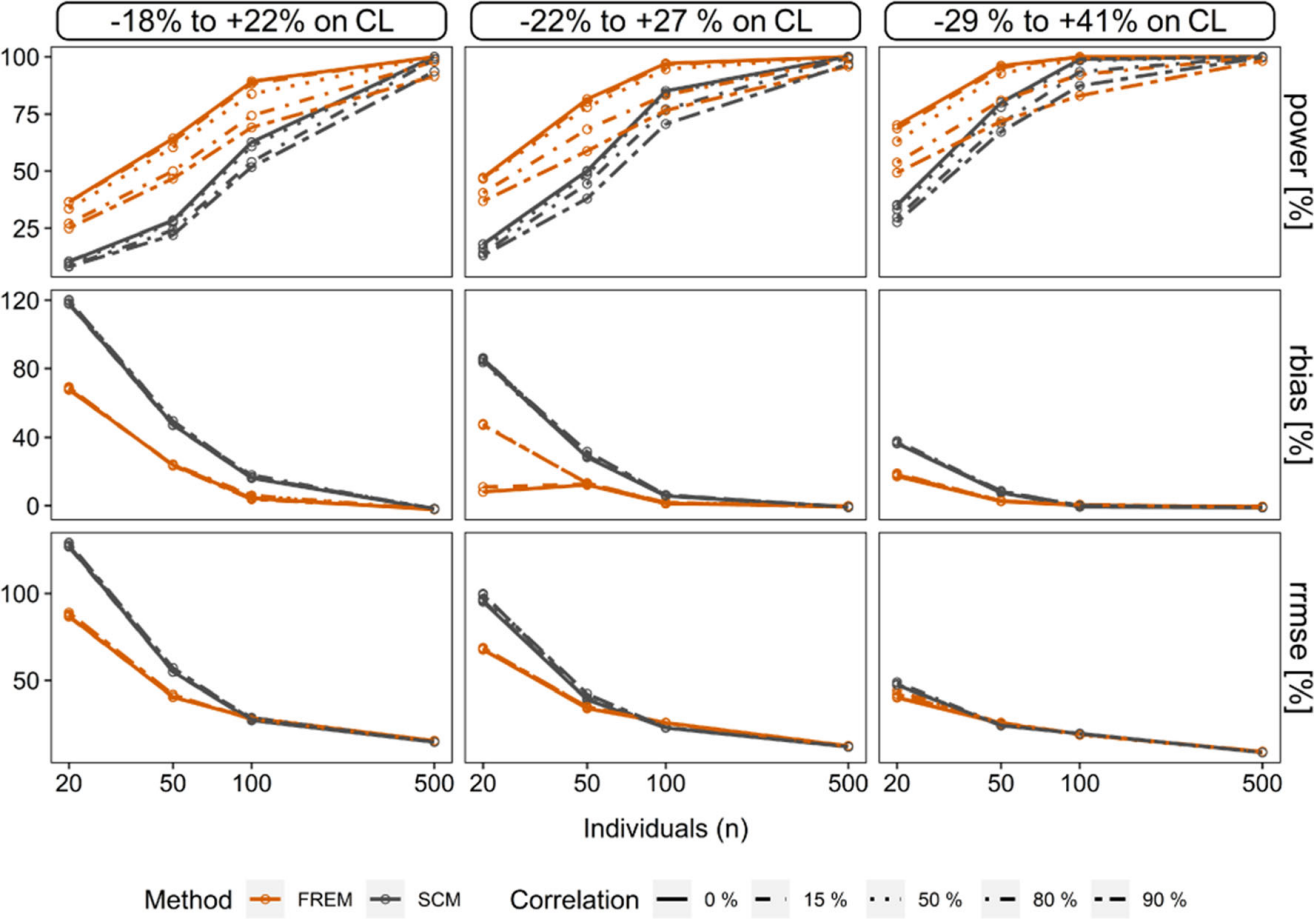
‘Scm’ and ‘frem_posthoc_’ results of scenario 1. Illustration of power (%), conditional relative bias (%) (*rbias*) and conditional relative root mean squared error (%) (*rrmse*) of *cov*_*true*_ estimates. Conditional accuracy and precision for the ‘frem_posthoc_’ is shown for the univariate coefficients

In scenario 1, the simulations and estimations showed that ‘frem_posthoc_’ power was higher compared to the ‘scm’ throughout all scenarios (Fig. 2), likely due to the higher value for alpha of 0.1 in the ‘frem_posthoc_’ (non-overlapping 90% confidence interval of the covariate effect size) vs. 0.01 in the ‘scm’. The dataset size (n = 20 to n = 100) strongly increased power for both methods. The presence of covariate correlation reduced the power of ‘frem_posthoc_’ from 82 to 59% (n = 50, $\theta_{cov_{true} }$ = 0.032) whereas the ‘scm’ power was less affected by correlation in the simulated scenarios (Table 1). Moreover, with an increasing covariate effect on clearance, we observed an increase of power from 28% (‘scm’, n = 50, 0% cov-corr, $\theta_{cov_{true} } = 0.026$) to 80% $(\theta_{cov_{true} } = 0.0{45)}$ and from 64 to 96% for ‘frem_posthoc_’. Scenarios with n = 500 showed a power of > 91%, independent of covariate effect magnitude and were less influenced by covariate collinearity (Fig. 2 and Table 1).

**Table 1:** Simulation and estimation results of ‘scm’ and ‘frem_posthoc_*’* in scenario 1

N	Covariate correlation (%)	Method	θ_COV_ = 0.026			θ_COV_ = 0.032			θ_COV_ = 0.045		
			power (%)	rbias (%)	rrmse (%)	power (%)	rbias (%)	rrmse (%)	power (%)	rbias (%)	rrmse (%)
20	0	frem	36.5	68.3	87.0	47.2	8.1	67.6	70.1	17.2	40.2
		scm	10.4	118	128	18.1	84.8	95.3	35.2	36.4	47.3
	50	frem	33.5	68.5	87.8	46.6	47.8	68.5	63.0	18.6	40.9
		scm	9.60	118	127	16.2	83.6	97.0	33.2	36.4	47.4
	90	frem	25.0	69.2	89.0	36.8	47.1	68.7	49.3	17.9	42.3
		scm	8.20	120	129	13.2	85.0	99.1	27.6	36.8	47.7
50	0	frem	64.2	23.8	40.5	81.5	12.5	33.9	96.0	2.50	25.3
		scm	28.4	47.3	54.9	50.1	28.3	39.0	80.0	7.60	24.2
	50	frem	60.4	23.7	40.6	78.0	12.8	34.4	92.6	3.00	25.2
		scm	27.5	47.2	54.8	48.4	28.8	39.6	78.0	8.00	24.2
	90	frem	46.5	23.4	41.8	58.8	13.2	34.9	71.8	2.70	25.8
		scm	22.1	49.5	57.1	38.0	31.6	42.2	67.2	8.30	24.2
100	0	frem	89.2	4.40	28.3	97.0	1.20	25.3	99.9	0.20	19.2
		scm	62.7	16.1	27.1	85.1	5.70	22.8	99.2	− 0.70	19.5
	50	frem	83.8	5.70	28.7	94.5	1.50	25.8	99.3	0.30	18.8
		scm	60.8	16.5	27.5	84.1	6.30	22.9	98.3	− 0.6	19.4
	90	frem	69.1	3.80	27.7	76.6	1.50	25.0	83.0	0.70	19.2
		scm	51.8	16.9	27.6	70.6	6.30	23.0	87.4	0.10	19.4
500	0	frem	99.2	− 2.2	15.4	99.9	− 0.2	12.4	100	− 0.50	8.80
		scm	100	− 2.1	14.6	100	− 1.0	11.9	100	− 1.2	8.60
	50	frem	99.2	− 2.3	15.3	100	− 0.2	12.4	100	− 1.0	8.70
		scm	100	− 2.0	14.6	100	− 1.0	11.9	100	− 1.1	8.60
	90	frem	91.96	− 1.8	15.1	95.8	− 0.7	12.1	98.1	− 1.0	9.10
		scm	93.5	− 1.7	14.6	96.7	− 0.8	11.9	99.6	− 1.1	8.60

The simulated relative covariate effect sizes were  − 18 to + 22% (θ_COV_ = 0.026),  − 22 to + 27% (θ_COV_ = 0.032) and  − 29 to + 41% (θ_COV_ = 0.045) on clearance at the 5th to 95th perc percentile of covariate values. Conditional accuracy and precision were expressed as rbias (%) and rrmse (%)

Moreover, the frequency of a significant cov_II_ effect in the final ‘frem_posthoc_’ models was > 77% in presence of ≥ 80% cov-corr (n ≥ 100). In contrast to that, cov_II_ was significantly included in < 16% of ‘scm’ runs.

**Fig. 3:**
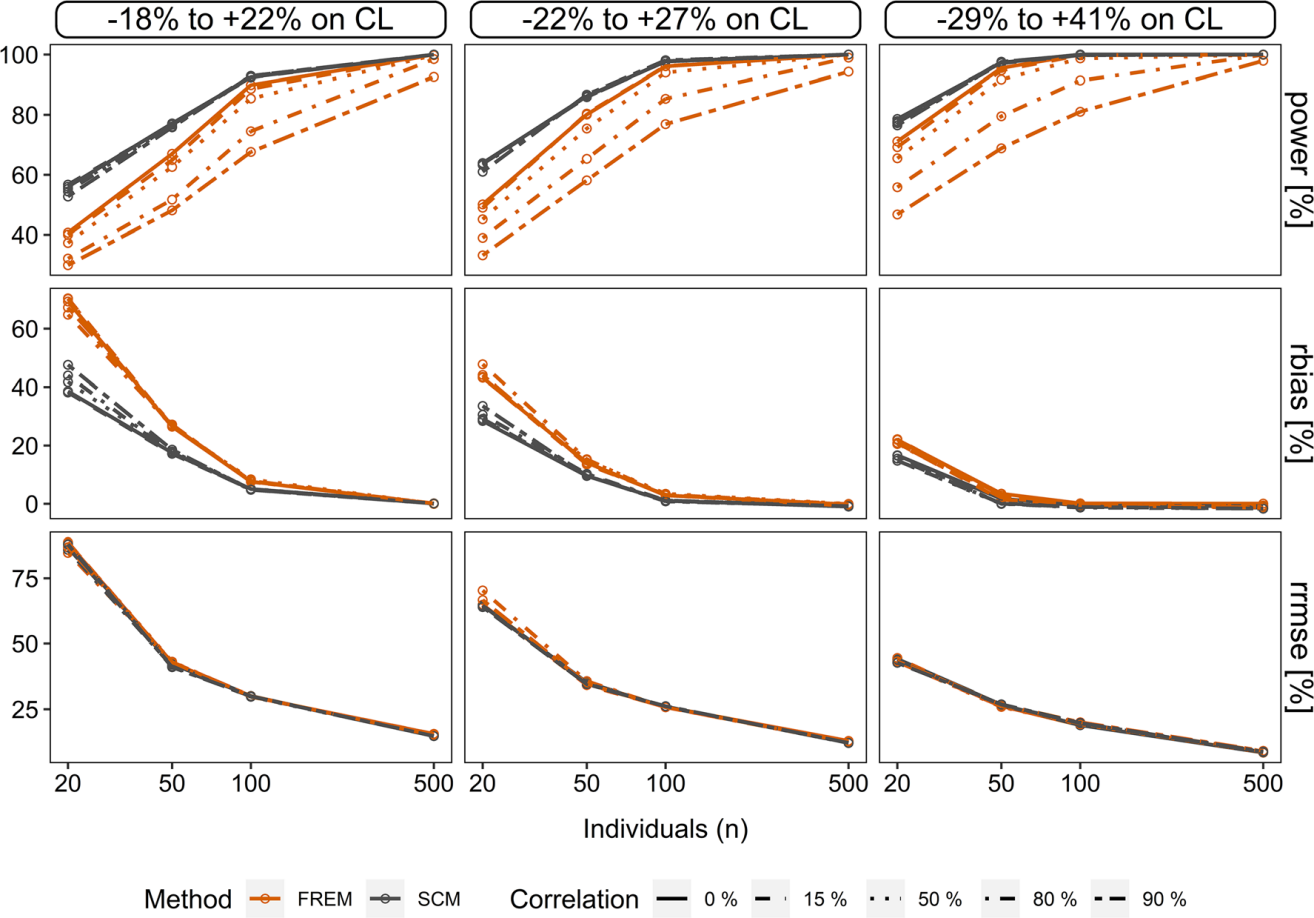
‘Scm’ vs. ‘frem_posthoc’_ in the scenario 2. Illustration of power (%), conditional relative bias (%) (*rbias*) and relative root mean squared error (%) (*rrmse*) of *cov*_*true*_ estimates in sparse datasets. Conditional accuracy and precision for the ‘frem_posthoc_’ is shown for the univariate coefficients

In scenario 2, a ‘*head-to-head’* comparison with a statistically similar setting was performed (same setting for the ‘frem_posthoc_’ as in scenario 1 and ‘scm’ with sole forward inclusion using an alpha value of 0.1): the less strict alpha value in combination with only forward inclusion led to an increase of power for the ‘scm’, resulting in above 53% and with that being superior to ‘frem_posthoc_’ (Fig. 3). More details are described in Supplement 3.

No comparison of power is possible for scenario 3 due to the missing selection step in the ‘frem’.

### Conditional accuracy and precision of $\theta_{cov_{true} }$ estimates

In scenario 1, an overestimation in small n datasets was more pronounced for ‘scm’ than for ‘frem_posthoc_’ (Fig. 2). Thus, ‘frem_posthoc_’ covariate coefficients were more accurate and precise. (Fig. 2, Supplement 3, Figure S3-2). We observed for both methods a power-dependent increase in conditional accuracy up to unbiased estimates, see Table 1 and Fig. 2. For example, the rbias of ‘scm’ coefficients was reduced from 50% ($\theta_{cov_{true} } = 0.026$) to 8% ($\theta_{cov_{true} } = 0.045$) in small datasets (n = 50) in presence of 90% cov-corr.

The conditional precision of the estimated coefficients in scenario 1 showed the same trend: Imprecision steeply decreased with increasing power (Table 1). With both methods, we obtained imprecise estimates in small n datasets (n = 50, $\theta_{cov_{true} } = 0.032$, ‘frem_posthoc_’: 35%, ‘scm’ 42%), independent of correlation.

In scenario 1, CL and V_c_ were accurately (rrmse < 10%) and precisely (rbias < 3%) estimated in the final ‘scm’ as well as the ‘frem_posthoc_’ model. The proportional error model estimate trended to underestimation (rbias >  − 11%) and was less precise with rrmse < 27%.

In scenario 2, the higher alpha value of 0.1 in scenario 1 for ‘scm’ forward selection strongly reduced overestimation of coefficients to a rbias below 48%. As a result, conditional accuracy was higher compared to ‘frem_posthoc_’, whereas conditional precision of ‘scm’ coefficients was similar to ‘frem’ coefficients throughout the scenarios (Fig. 3, Supplement 3 Table S3-1). Additional details are described in Supplement 3.

**Fig. 4:**
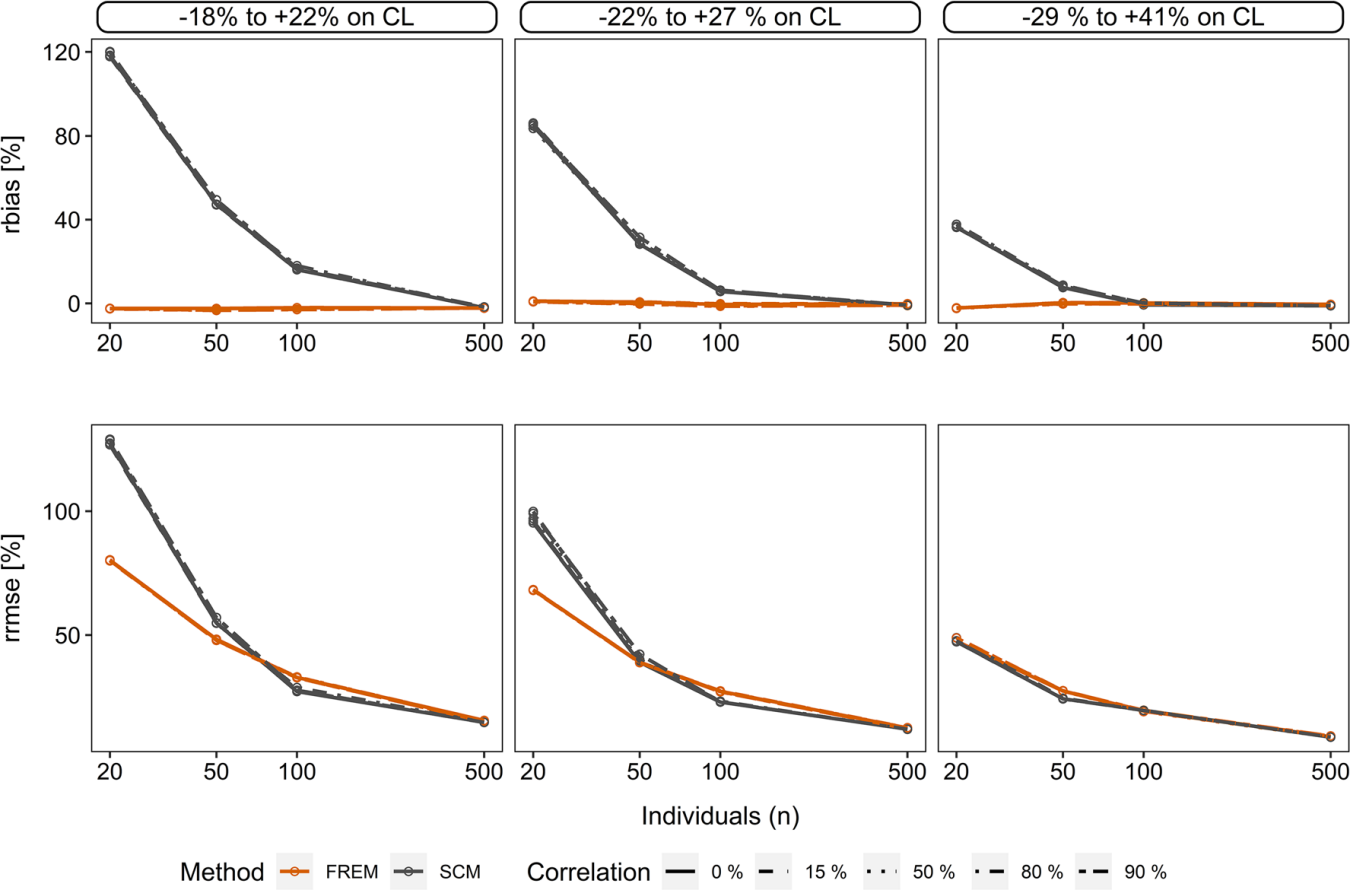
**‘**Scm’ vs. ‘frem’ for scenario 3. Illustration of relative bias (%) (*rbias*) and relative root mean squared error (%) (*rrmse*) of *cov*_*true*_ estimates in sparse datasets. Accuracy and precision for the ‘frem’ is shown for the univariate coefficients

Furthermore, scenario 3 compared all ‘frem’ cov_true_ estimates without a selection step to those of the final ‘scm’ models obtained after backward elimination. This analysis quantitatively shows the effect of selection bias if compared to scenario 1 results. In sum all ‘frem’ coefficients were unbiased. Moreover we observed still a high imprecision of ‘frem’ coefficients in small n datasets (n < 100) which was independent of the selection step, but ‘frem’ showed a superior precision compared to ‘scm’ especially in small n datasets, (Fig. 4). Further details are described in Supplement 3.

The simulation study using a true categorical covariate showed the same trend of power, conditional rbias and rrmse for scenario 1 and scenario 2, whereas the differences of our evaluation criteria were smaller between ‘scm’ and ‘frem_posthoc_’, if compared to the simulation study using a true continuous covariate. Supplement 2 provides a detailed description of all obtained results.

### Predictive performance of ‘scm’ and ‘frem_posthoc_’ models

**Fig. 5:**
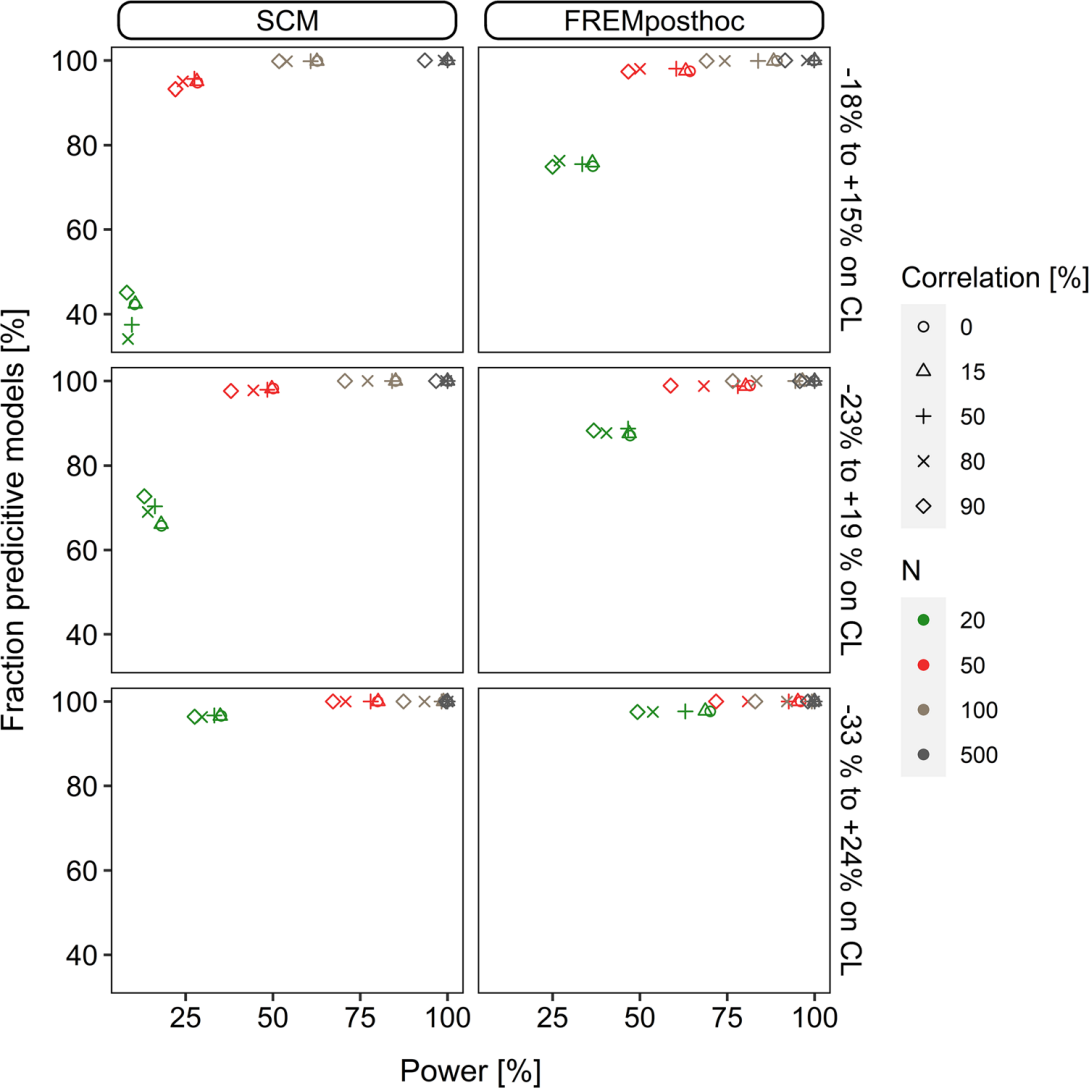
Fraction of models with high predictive performance for ‘scm’ and final ‘frem_posthoc_’ models with significant true covariate relationships in scenario 1. Estimated coefficients between zero to two times the true value were assumed to improve the predictive performance

Scenario 1 evaluated the estimated covariate coefficients of the final ‘scm’ and ‘frem_posthoc_’ models for their predictivity, i.e., were termed predictive when estimated between zero and two times the true value. The results are shown in Fig. 5. The predictive performance of the cov_true_ estimates was a function of power for the ‘scm’, but also for ‘frem_posthoc_’. The ‘frem_posthoc_’ showed a higher power in small n datasets, thus the fraction of predictive models was more than twice as high compared to ‘scm’. On the other hand, the fraction of predictive ‘scm’ models increased more steeply with increasing power. At a power value of > 28% more than 90% of the final models were likely to improve the predictivity (‘frem_posthoc_’ > 47% power).

As power is a composite of dataset size, covariate effect size and correlation, we analysed the individual components on their relation to influence the fraction of predictive models (Supplement 3 Figure S3-3). We observed that dataset size, covariate effect size, rbias and rrmse most influenced the fraction of predictive models and that predictive performance was less impacted by covariate correlation.

The fraction of predictive ‘scm’ and ‘frem_posthoc_’ models in scenario 2 were similar (scm: 97.0% ‘frem_posthoc_’: 97.5%, n = 50, cov-corr = 80%, $\theta_{cov_{true} } = 0.026$) and reached both 100% in the scenario with the highest simulated covariate effect magnitude, $\theta_{cov_{true} } = 0.045,$ n > 50), see Supplement 3 Figure S3–4.

Overall, final ‘frem’ models (scenario 3) were providing highly predictive covariate coefficient estimates, which were mainly driven by covariate effect magnitude and independent of the dataset size (Supplement 3 Figure S3-6).

### Type 1 error

**Fig. 6:**
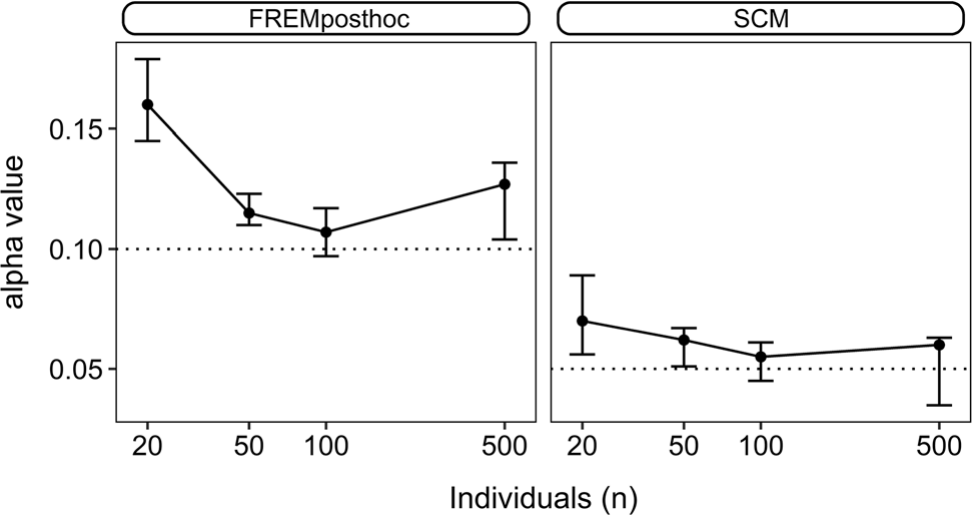
True alpha-values for ‘frem_posthoc_’ and ‘scm’ for scenario 1. Error bars shows min and maximum values, and points display median values

For scenario 1, the true alpha values are displayed in Fig. 6. Overall, ‘frem_posthoc_’ indicated a false significant covariate effect of the dummy covariate cov_III_ in more cases, than the given 10% confidence intervals of the covariate effects would imply, i.e., an inflated type 1 error rate was observed. The ‘scm’ also displayed inflated type 1 error rates for small datasets. For n ≥ 100 both methods approached the set alpha value of 10% (‘frem_posthoc_’) or 5% (‘scm’).

In scenario 2, the true ‘scm’ alpha values were between 6 and 11% and with that close to the expected 10% value.

## Discussion

In the present study, we compared operational characteristics of the novel ‘frem’ technique to ‘scm’ as automated covariate analysis methods. As the ‘frem’ method is a full model approach and does not originally comprise a selection step, we introduced the ‘frem_posthoc_’ step to account for a covariate backward elimination based on significant covariate effect sizes. This reflects an additional application of a ‘frem’ model in an exploratory analysis. Overall, this study gave insights in operational characteristics of the ‘frem’ method, but also showed the ability of ‘frem_posthoc_’ to guide covariate selection. Yet, for ‘frem_posthoc_’ the same caution as for the ‘scm’ should be applied since this posthoc step also can introduce selection bias in scenarios with low power (i.e. small covariate effect size, small sample size). Of note, an evaluation of precision and accuracy of all cov_true_ ‘frem’ estimates without a selection step showed that the covariate effect estimates were unbiased and showed lower imprecision as those determined using the ‘scm’, which were biased due to the selection step, in particular in scenarios with low power. This underlines the value of the ‘frem’ method. It has the additional advantage of interpreting the covariate effect simultaneously to statistical significance without the need for further evaluate the parameter uncertainty, which is needed for ‘scm’ to evaluate clinical relevance (e.g. bootstrap, llp-sir [17]). In large datasets, both methods provided precise and accurate inference on covariate effects in our simulation study. Moreover, Yngman et al. described an advantage of ‘frem’ model, that it can provide covariate coefficients for any subset of the examined covariates and thus be applied to different covariate datasets [7]. In addition, a model reduction of the full model could be done in a stepwise manner, if a more parsimonious model is desired [2, 7]. This simulation study comprised an investigation of final ‘frem’ model subsets for the purpose of covariate backward elimination, presented in scenarios 1–2.

The statistical power to detect true covariate effects is important to guide clinical study design. Ribbing et al. described that dataset size, magnitude of collinearity, and covariate effect size influence the power of the ‘scm’ method [16]. Ahamadi et al. investigated the operating characteristics of ‘scm’ using different complexities of true models (i.e. 1–4 true covariates). Those scenarios with one true covariate (n = 300, cov-corr 32% or 89%, 250 simulations) reached a high power [18]. This is in line with our results in datasets n ≥ 100. Beyond that, our observed power increase, as a result of increased dataset- and covariate effect size, as well as a reduction of power caused by collinearity of covariates are in line with Ribbing et al. [19]. In comparison to that, the ‘frem_posthoc_’ showed an up to three-fold higher power in the worst-case scenario with high correlation in small cohort studies (scenario 1, $\theta_{cov_{true} } = 0.026$), likely as a result of the different alpha values in the selection step. In scenario 2, power differences of the two methods were smaller, rather favouring ‘scm’. We however think that ‘scm’ with only forward inclusion and an alpha value of 0.1 does not represent common practice. Moreover, it is known that ‘scm’ suffers from multiple testing which is not the case of the ‘frem_posthoc_’ method, which makes this an interesting comparison.

In this study cov_II_ carries up to 90% of the information of cov_true_. ‘Frem_posthoc_’ accounts for correlation and the high frequency significant cov_II_ inclusions in high correlation scenarios represents its ability to account for correlation. In contrast to that, ‘scm’ with forward selection (p value < 0.05) and backward elimination (p value < 0.01), but also with applying only forward inclusion (p-value < 0.1) is intrinsically not able to capture the true present correlation. However, the model prediction using a wrong, but highly correlated covariate, that carries information of the true covariate could be comparable to including the true covariate. One the one hand, the inclusion would lead to interpretation difficulties, on the other hand, an exclusion of correlated covariates could also cause confounded interpretation of covariate effect estimates, as the correlated covariate carries parts of the true covariate information. Thereby pharmacological understanding is key for decision making.

We also investigated a scenario with a true categorical covariate with and without an additional level of variability, the IIVV_c_. The results showed a similar behaviour as observed for continuous covariates. Scenarios 1 and 2 showed only minor differences in power for ‘scm’ and ‘frem_posthoc_’ in cases when the covariate has a strong effect size. The additional level of variability decreased power by up to ca.  − 5%. The simulation study using a true continuous covariate did not include IIVV_c_, so we assume an overall worsening effect of the presented continuous covariate study results in presence of IIVV_c_ here.

Moreover, conditional accuracy and precision of the covariate coefficients were investigated in case cov_true_ was selected in the final models. In scenario 1 bias was present in both methods, however slightly lower when using the ‘frem_posthoc_’ (especially in low power scenarios). In scenario 2 the findings were vice versa, so that overestimation was less pronounced for ‘scm’, resulting from a less strict alpha value in the selection step. According to Wahlby et al. selection bias is only moderate in typical PK modelling dataset [5], but this was only confirmed for covariates with high effect sizes [16]. In scenario 3 unbiased ‘frem’ estimates were obtained, as no covariates were selected, and all estimated coefficients were considered for the evaluation.

Conditional precision was more precise for ‘frem_posthoc_’ compared to ‘scm’ in scenario 1 and equally high in scenario 2. Precision was improved by a less strict alpha value (‘scm’ in scenario 1 vs. scenario 2). As precision is a function of power, we assume that the increased precision is caused by increased ‘scm’ power.

Beyond that, in scenario 1 we evaluated the predictive performance of the final models and used the range of zero to two times the true coefficient value as a predictor for improvement of the model fit, according to Ribbing et al. [16]. The present study confirmed the predictive performance of ‘scm’ models being a function of power and we confirmed this for ‘frem_posthoc_’ estimates. Compared to ‘scm’ models, the fraction of predictive ‘frem_posthoc_’ models was higher, especially in scenarios which achieved power < 50%. The predictive performance was positively correlated with rbias, rrmse and number of study individuals. Interestingly, covariate collinearity did not impact the predictive performance (Supplement 3 Figure S3-3).

The type 1 error rate was evaluated with cov_III_ being independent from the other two available covariates. The previously described inflated type 1 error rate in the ‘scm’ approach [5] was confirmed in this study but was also observed for the ‘frem_posthoc_’. The ‘frem_posthoc_’ alpha values were decreasing with increasing study size but were still slightly inflated. The confidence interval of the covariate effect is calculated by SIR in the PsN implementation of ‘frem’ [15]. The confidence interval served for the calculation of the frequency in how many of the performed runs the cov_III_ effect size was estimated to be significantly different from zero. Broeker et al. found, that especially in small n datasets the SIR-derived confidence interval tends to be underestimated, in particular for the omega values [17]. This underestimation might explain the inflated alpha values, as zero is less often included in the SIR-based confidence intervals if they are too narrow.

‘Frem’ is mathematically equivalent to FFEM, which has been suggested as an alternative to stepwise procedures [2]. Although a backward elimination is not originally intended by the full model approach, as this may curtail its benefits, a guidance for this backward elimination step has been proposed by Gastonguay et al. [2]. A model reduction based on covariate effect size, has also been applied to clinical data [20]. A reduction of a full model for predictive purposes can be done via exclusion of non-statistically significant (CI includes null value) and non-clinically important (entire CI contained within no effect range) covariate effects. Covariates which are clinically important and statistically significant, or are not statistically significant but may be clinically important should be retained in the model [2]. The clinical relevance criteria was not considered in our study evaluation, as this additional filter is subjective in a simulation study and driven by the pharmacological considerations. Furthermore, statistically significant effects are clearly defined, whereas the often used clinical relevance threshold of 20% is not. This threshold may apply for clearance; however, it can be different for other PK parameters related to a covariate effect. Moreover, this threshold can be dependent on the indication, pharmacometric question to be answered or substance itself, e.g. a narrow therapeutic window could reduce the threshold. These factors cannot be fully reflected in a simulation study.

A few more limitations shall be mentioned: the here evaluated scenarios only display a portion of the complexity of covariate analysis in real clinical datasets. The here simulated cov_true_ effect magnitudes were chosen around the often-used clinical significance threshold of 20% on clearance [12] displaying a weak, moderate, and strong effect as it could be expected in a real clinical dataset. However, neither collinearity between more than one covariate, nor the presence of more than one true covariate carrying information was investigated.

To calculate the fraction of predictive models amongst the evaluated runs in each scenario, we assumed an estimated covariate coefficient between zero and two times cov_true_ to be likely to improve the predictive performance of a model [16]. This in other words accounts for up to 100% overestimation, so that even in presence of a strong selection bias, coefficients were rated as predictive. Highly biased covariate coefficients make the model less adequate for predictive purposes and could ultimately cause misleading clinical interpretation on e.g., clearance if the covariate coefficient originates from small n datasets (< n = 100). However, as ‘frem_posthoc_’ has not been applied to clinical data yet, this needs to be further evaluated.

Besides that, this simulation study investigated only covariates on clearance, but usually clinical covariates are also found on other model parameters. Moreover, interindividual variability on central volume of distribution is very common in clinical datasets but was not included in the analysis using a true continuous covariate. Based on prior knowledge and confirmatory results obtained in the simulation study with categorical data, we assume a reduction of power in presence of more levels of variability. Moreover, the covariate coefficients directly obtained via the PsN ‘frem’ routine, represent exponential covariate parameterization in fixed effects models [8]. Other implementations might be of interest, too, and could be explored in subsequent studies.

## Conclusion

Overall, this study contributed to the understanding of the ‘frem’ and showed properties and characteristics of the methods for continuous but also categorical covariates. We introduced with ‘frem_posthoc_’ a possibility to guide covariate selection, mimicking how ‘frem’ could be additionally used in practise. With that, covariate effect size interpretation and selection can be done simultaneously and a predictive model with capturing correlation in the datasets can be obtained. Using the commonly applied settings of ‘scm’ and ‘frem’, in small n datasets the power of ‘frem_posthoc_’ was substantially higher, leading to a lower bias, compared to ‘scm’ in scenario 1. In datasets with n > 100 power, precision, and accuracy of ‘frem_posthoc_’ were comparable to ‘scm’. However, the simulated scenarios still highlight the need for thoughtful choice of the method to answer the underlying pharmacometric question in small datasets.

## Supplementary Information

Below is the link to the electronic supplementary material.

Supplementary file1 (DOCX 66 kb)

Supplementary file2 (DOCX 1059 kb)

Supplementary file3 (DOCX 2671 kb)
