# Supplementary material for: Operational characteristics of full random effects modelling (‘frem’) compared to stepwise covariate modelling (‘scm’)
Source: J Pharmacokinet Pharmacodyn. 2023 Apr 21;50(4):315–26. doi: 10.1007/s10928-023-09856-w (PMC10374720; doi:10.1007/s10928-023-09856-w)
Supplement: Supplementary file 1 — Supplementary file1 (DOCX 66 kb) [file 10928_2023_9856_MOESM1_ESM.docx]

**Supplement 1**

1. **Run001,true one compartment model with i.v. infusion and linear pharmacokinetics including the true covariate relationship on clearance.**

;; 1. Based on: --

;; 2. Description: Model with true covariate relationship

;; x1. Author: user

;; 3. Label: -

$PROBLEM SIM

$INPUT

ID

TIME

DV

AMT

RATE

EVID

MDV

CMT

OCC

COV1

COV2

COV3

$DATA sim_temp.csv IGNORE=@

$SUBROUTINE ADVAN1 TRANS2

;----------------------------------

$PK

;FREELINE1;

;FREELINE2;

;FREELINE3;

;FREELINE4;

TVCL = THETA(1)* EXP(THETA(5)*(COV1- XXX )) ; will be replaced in R by the mean of simulated cov values

CL = TVCL*EXP(ETA(1))

TVV1 = THETA(2)

V = TVV1

KE = CL/V

S1=V

$ERROR

IPRED = A(1)/V

W = SQRT( THETA(3)**2*IPRED**2 + THETA(4)**2)

Y = IPRED + W*EPS(1)

IRES = DV-IPRED

IWRES = IRES/W

$THETA

(18) FIX ; 1_CL

(400) FIX ; 2_V

(0.15) FIX ; 3_proportional error

(0.001) FIX ; 4_add. Error

(0.026) FIX ; 5_COV1 ,three3 mod files for all different covariate effect size scenarios (0.032, 0.045)

;FREELINE5; ; Placeholder

;FREELINE6;

$OMEGA

0.1 FIX ; 1_IIV_CL

$SIGMA 1 FIX ;

$SIM(20101994) (1234) ONLYSIM ;Seed will be changed with R

NSUBPROBLEMS=1

;XPOSE4-----

$TABLE ID TIME AMT PRED IPRED RATE IPRED EVID MDV CMT CL V ETA1 OCC COV1 COV2 COV3 WRES CWRES NPDE ONEHEADER NOPRINT FILE=sdtab001

1. **Run002, the structural base model without a covariate relationship serving for ‘scm’ and ‘frem’ executions**

;; 1. Based on: -

;; 2. Description: STRUC BASE MODEL CORR15 FOR SCM FREM

;; x1. Author: user

;; 3. Label: -

$PROBLEM SCM vs FREM

$INPUT ID

TIME

DV

AMT

RATE

EVID

MDV

CMT

OCC

COV1

COV2

COV3

$DATA cov_temp.csv IGNORE=@ IGNORE(ID>100) ; correct number of IDs adjusted via R code

$SUBROUTINE ADVAN1 TRANS2

;----------------------------------

$PK

;FREELINE1; ; Placeholders

;FREELINE2;

;FREELINE3;

;FREELINE4;

TVCL = THETA(1)

CL = TVCL*EXP(ETA(1))

TVV = THETA(2)

V = TVV

KE = CL/V

S1=V

PROP = THETA(3)

ADD = THETA(4)

;----------------------------------

$ERROR

IPRED = A(1)/V

W = SQRT( THETA(3)**2*IPRED**2 + THETA(4)**2)

Y = IPRED + W*EPS(1)

IRES = DV-IPRED

IWRES = IRES/W

;----------------------------------

$THETA

(0,21) ; 1_CL

(0,400) ; 2_V

(0,0.2) ; 3_proportional error

(0.001) FIX ; 4_add. Error

;FREELINE5; ; Placeholder

;FREELINE6;

$OMEGA

0.2 ; 1_IIV_CL

$SIGMA 1 FIX

$EST METHOD=1 INTER MAXEVAL=9999 PRINT=20 NOABORT SIGL=3

$COV

$TABLE ID TIME MDV AMT EVID OCC CL V PROP ADD PRED ETA1 IPRED IWRES CWRES NPDE ONEHEADER NOPRINT FILE=sdtab002

;XXTABLEXX ; Placeholder to adjust output table via R

1. **SCM configuration file**

;;config_template_standard.scm

;;lines starting with ; are comments

;;if a line starts with ; it must not end with \ because that will cause

;;strange errors.

;;Some of the options in this file can also be given on the command-line,

;;but it is convenient to set them in the config file

;;Most of the options in this file are optional, but this file is a good

;;check-list. Not all scm options are listed here, see the userguide for

;;a complete list.

;;Edit as needed, comment/uncomment options to suit your run

;;model file without any of the covariate combinations in test_relations.

;;Other covariates may be included

;;There is no run34.mod in the extra material

model=run002.mod

;;search direction can be forward, backward or both

;;to be adjusted in case of 'head to head' comparison

search_direction=both

;;provided that the perl module Math::CDF is installed, any p-values can

;;be used. p_backward should be smaller than p_forward

;; backward elimination was not present in 'head to head' comparison

p_forward=0.05

p_backward=0.01

;;it is required to list the covariates to test

continuous_covariates=COV1,COV2,COV3

;;By default option parallel_states=0 and scm tries parameterizations

;;one at a time, in the order set in valid_states. Only if the covariate

;;is included in the model with the first parameterization is the next

;;parameterization tried.

;;if parallel_states is set to 1, scm will test all possible relation

;;forms for a parameter-covariate pair simultaneously.

parallel_states=1

;;These general PsN options that can be set in the configuration file.

;;Most general PsN options must however be set on the command-line

retries=3

threads=1

tweak_inits=1

picky=0

;;In the configuration file all single-line options must come BEFORE the

;;first bracket-header section, otherwise the options will be ignored by scm

;;Each bracket-header section can have many lines, but each header must

;;appear at most one time

[test_relations]

CL=COV1,COV2,COV3

;;valid_states (possibly in combination with [code]) tells scm which

;;parameterizations should be tested for the covariates

;;There are default meanings to numbers 1-5, but by adding a [code] section

;;new parameterizations can be defined, and numbers can be set to mean

;;different parameterizations for different parameter-covariate pairs

;;The first valid state must always be 1

[valid_states]

continuous = 1,4

categorical = 1,2

1. **R code for clinical dataset generation and study scenario creation, using run001 for simulations and run002 for ‘scm’ and ‘frem’ executions.**

#-clear environment

rm(list=ls())

#-read in packages

library(data.table)

library(tidyverse)

require(zoo)

require(boot)

require(xpose4)

library(dplyr)

library(foreach)

library(doParallel)

library(tidyr)

library(MASS)

library(janitor)

library(stringr)

###############################################################

# CREATE DATASET FUNCTION #

#'##############################################################

create.workingdata = function(regimen, observations, covariates.const,

uncertainty.time.sd, # additive sd in h - not used in this study

uncertainty.RATE.sd, # additive sd in h - not used in this study

n_ID){ # number of IDs to simulate

#-combine dataset components into a list

input.list = list(regimen, observations)

#-eliminate NULL items before merge (required)

input.list = input.list[which(!sapply(input.list, is.null))]

dummy_out = NULL

for (i in 1:n_ID) {

#-merge regimen, observations and covariates

dummy = Reduce(function(...) merge(..., all = TRUE, by = "TIME"), input.list)

dummy = as.data.table(dummy)

dummy$EVID = 0

dummy$EVID[dummy$AMT > 0] = 1

# dummy[dv >= 0, EVID := 0]

#-add uncertainty to sampling TIME if needed (not used in this study)

dummy$TIME[dummy$EVID == 0] = dummy$TIME[dummy$EVID == 0] +

rnorm(n = length(dummy$TIME[dummy$EVID == 0]), mean = 0, sd = uncertainty.time.sd)

dummy$RATE[dummy$EVID == 1] = abs(dummy$RATE[dummy$EVID == 1] +

rnorm(n = length(dummy$RATE[dummy$EVID == 1]), mean = 0, sd = uncertainty.RATE.sd))

dummy$RATE = dummy$AMT / (dummy[,"RATE",with = F])

dummy$RATE[is.na(dummy$RATE)] = 0

dummy$AMT[is.na(dummy$AMT)] = 0

dummy$ID = i

dummy$CMT = 1

dummy = dummy[order(TIME),]

dummy[EVID == 1, OCC := seq(1,length(dummy[EVID == 1]$TIME),by=1)]

dummy[,OCC := na.locf(OCC)]

dummy$MDV = 1

dummy$MDV[dummy$EVID == 0] = 0

dummy_out = rbind(dummy_out, dummy)

}

return(dummy_out)

}

################################################################

# CREATE WORKING DATASET #

#'###############################################################

#-define max. number of IDs in dataset

n_ID = 500

#-define number of simulations

n_sim = seq(from = 0, to = 1000, by =1)

#-create seed numbers

seed=round(runif(1000,min=10000,max=99900),digits=0)

#-define dosing regimen

regimen1 = data.frame(TIME = c(0,12,24,36,48,60,72,84,96,108,120),

AMT = c(100, rep(50,10)), RATE = c(0.5))

#-define timepoints of observations

#-per ID one trough sample, one sample 1h after peak concentration

observations1 = data.frame(TIME = c(71.5, 109), DV = NA )

covariates.const1 = NULL

###################################################################

# LOOP STARTS #

###################################################################

#-create lists for collecting simulation study data in loop

rep_list_data = vector("list", length(n_sim))

rep_list_pop = vector("list", length(n_sim))

frem_evaluation_out = vector("list", length(n_sim))

#-define covariate correlation, as well as dataset size (n) for the simulation study

correlation = c(0, 0.15,0.5, 0.80, 0.90)

IDs =c(20,50,100,500)

#-define cores for parallelisation

n_cores=20

for( j in IDs){

for (z in correlation) {

print(j, z)

#-register on cluster for parallelisation of the n_simulation loop

cl = makeCluster(n_cores)

registerDoParallel(cl)

#-start parallelization

simulations = foreach (a = unique(n_sim), .errorhandling = "pass", .verbose = T) %dopar% {

library(data.table)

require(zoo)

require(xpose4)

library(dplyr)

library(tidyr)

library(MASS)

library(janitor)

library(stringr)

require(boot)

#-create dataset for the scenario according to loop

dataset1 = create.workingdata(regimen = regimen1,

observations = observations1,

covariates.const = covariates.const1,

uncertainty.time.sd = 0, # no uncertanty

uncertainty.RATE.sd = 0, # no uncertanty

n_ID = n_ID)

# “.” for DV

dataset1[,DV := as.character(DV)]

dataset1[,DV := "."]

dataset1$RATE[is.na(dataset1$RATE)] = "."

#-setting sample size for multivariate normally distributed samples

N <-500

#-setting the means of covariates

mu <- c(28,8, 5.8)

#-create covariate-correlation matrix (3x3 matrix)

#-desired variances: 15,1.2,0.3 of the covariates

#-z defines correlation between covariate 1 and 2

sigma <- matrix(c(15, z*sqrt(15*1.2), 0,

z*sqrt(15*1.2), 1.2,0,

0,0, 0.3),3,3)

#-setting the seed value

set.seed(seed[a])

#-simulate the data, as specified above

df1 <- mvrnorm(n=N,mu=mu,Sigma=sigma)

#-extract simulated covariate values

COV1 = as.data.frame(df1)

#-create covariate dataset

data = data.frame(ID=c(1:n_ID))

data$COV1 = COV1$V1

data$COV2 = COV1$V2

data$COV3 = COV1$V3

data_out = left_join(dataset1,data, by="ID")

data_out = dplyr::select(data_out, ID, TIME, DV, AMT, RATE, EVID, MDV, CMT, OCC, COV1, COV2, COV3)

#-provide simulated dataset for NONMEM simulation with "true model" to obtain DV values

#-each dataset is individual for the respective scenario and simulation

write.csv(data_out, paste("Datasets_SIM/sim_temp", j, "IDs", z*100, "corr_sim", a, ".csv", sep= "" ) ,

row.names = F, quote = F)

##################################################################

# SIM WITH TRUE MODEL #

###################################################################

#-read in "true model" modfile

modfile = scan("run001.mod", sep = "\n", what = character(),

quiet = TRUE)

#-change seed

seed_n_sim=seed[a]

modfile_train = gsub("20101994", as.character(seed_n_sim),modfile, ignore.case=T)

#-normalize to mean of covariate (as automated in FREM)

modfile_seed_train = gsub("XXX", round(mean(COV1$V1), digits=3) , modfile_train, ignore.case=T)

#-navigate to simulated dataset

modfile_seed_train[19] = paste("$DATA Datasets_SIM/sim_temp",j, "IDs", z*100, "corr_sim", a, ".csv IGNORE=@ ",

" IGNORE(ID >",j, ")", sep = "")

#-Create unique sdtabs for each execution with NONMEM simulations

modfile_seed_train = gsub("FILE=sdtab001", as.character(paste("FILE= sdtab_train_", j, "IDs_", z*100,"corr_sim", a, sep = "")), modfile_seed_train, ignore.case=T)

#-save changes in "true model"

write(modfile_seed_train, file = paste("run001seed_train_",j, "IDs_", z*100, "corr_sim", a, ".mod" , sep = ""))

#-execute NONMEM model for simulations

system(paste("execute ", "run001seed_train_",j, "IDs_", z*100, "corr_sim", a, ".mod", " -model_dir_name -silent

-clean=3", sep = ""), wait = T, intern = F)

#-read in simulated sdtab

#-dataset for FREM and SCM runs for training

dataset1.sim_train = read.table(paste("sdtab_train_", j, "IDs_", z*100,"corr_sim", a, sep = ""),skip = 1, header = T)

#-copy simulated DV into simulated dataset1 and filter dataset size according to j

dataset2 = copy(data_out) %>% dplyr::filter(ID <= j)

dataset2[,DV := dataset1.sim_train$DV[dataset1.sim_train$ID <= j]]

dataset2$DV[dataset2$MDV == 1] = "."

#create "clinical dataset" including DV and covariates for use in scm and frem executions

write.csv(dataset2,paste("Datasets_SIM/cov_temp", j, "IDs", z*100, "corr_sim", a, ".csv", sep= "" ) ,

row.names = F, quote = F)

###################################################################

# START SCM AND FREM #

###################################################################

#-read in structural base model to change it individually according to the study scenario

run002 = scan("run002.mod", sep = "\n", what = character(), quiet = TRUE)

#-provide clinical dataset and edit IDs according to the scenario

run002[20] = paste("$DATA ", "Datasets_SIM/cov_temp",j, "IDs", z*100, "corr_sim", a, ".csv", " IGNORE=@

IGNORE(ID>", j, ")", sep = "")

#-save changes in mod file

write(run002,file = paste("run002_",j, "IDs_", z*100, "corr_sim", a, ".mod", sep = ""))

#-change scm configuration file according to the study scenarios (change of IDs and modfile name)

scm.file = scan("scm_run002.scm", sep = "\n", what = character(), quiet = TRUE) # edid SCM config file

scm.file = gsub("model=run002.mod", as.character(paste("model= run002_",j, "IDs_", z*100, "corr_sim", a, ".mod",

sep = "")), scm.file)

write(scm.file, file = paste("scm_run002_",j, "IDs_", z*100, "corr_sim", a, ".scm", sep = ""))

#-execute structural base model (without a covariate relationship)

system(paste("execute ", "run002_",j, "IDs_", z*100, "corr_sim", a, ".mod", " -model_dir_name -min_retries=3 -silent -

clean=3",sep =""), wait = T, intern = F)

#-start SCM

system(paste("scm ", "run002_",j, "IDs_", z*100, "corr_sim", a, ".mod", " -dir=SCM_RUN_",j, "IDs_", z*100, "corr_sim",

a, " -silent -config_file=scm_run002_",j, "IDs_", z*100, "corr_sim", a, ".scm -clean=3", sep =""),

wait = T, intern = F)

#-start FREM

system(paste("frem ", "run002_",j, "IDs_", z*100, "corr_sim", a, ".mod", " -dir=FREM_RUN_", j, "IDs_", z*100,

"corr_sim", a, " -silent -covariates=COV1,COV2,COV3 -run_sir -check -rplots=2 -clean=3", sep =""),

wait = T, intern = F)

#-check if frem output was completely created - if not delete and restart – manual "retries” of frem method

x = 1

while(length(list.files(path = paste("FREM_RUN_", j, "IDs_", z*100, "corr_sim", a, sep=""),

pattern = "*.html")) == 0 && (x <=3 )){

x= x+1

print(x)

#-delete not successful frem run and start new one with same directory

system(paste("rm -r FREM_RUN_", j, "IDs_", z*100, "corr_sim", a, sep=""), wait = T, intern = F)

system(paste("frem", " run002_",j, "IDs_", z*100, "corr_sim", a, ".mod", " -dir=FREM_RUN_", j, "IDs_", z*100,

"corr_sim", a, " -silent -covariates=COV1,COV2,COV3 -run_sir -check -rplots=2 -clean=3", sep =""),

wait = T, intern = F)

}

#-Print information about status of simulation estimation via Rout data

print(paste("SCM FREM Cycle", dir," completed at", Sys.time()))

}

stopCluster(cl) #stop parallelisation for that loop

} #-close ‘correlation’ loop

} #-close ‘ID’ loop

###################################################################

# END OF EXECUTIONS #

###################################################################

1. **R code for ‘frem’ results extraction**

#-clear environment

rm(list=ls())

#-load R packages

library(data.table)

library(tidyverse)

library (zoo)

library (boot)

library (xpose4)

library(stringr)

library(janitor)

#-define scenarios

IDs = c(20,50,100,500)

CORR = c(0,0.15,0.5,0.8,0.9)

SIM = seq(1,1000)

#-create folder names to navigate into frem folders

loop1 = list()

loop2 = list()

loop3 = list()

for( i in IDs){

for(c in CORR){

for(s in SIM){

loop1[[s]] = paste("FREM_RUN_",i, "IDs_", c*100, "corr_sim", s, sep ="")

}

loop2[[which(CORR == c)]] = as.character(unlist(loop1))

}

loop3[[which(IDs == i)]] = as.character(unlist(loop2))

}

fremruns = unique(as.character(unlist(loop3)))

#################################

# FREM ANALYSIS #

#################################

#-prepare lists for collection of frem output results

rep_list_coeff = list()

rep_list_cov = list()

rep_list_pop = list()

rep_list_coeff.loop = list()

rep_list_cov.loop = list()

rep_list_pop.loop = list()

#-directories for covariate effect size scenarios. For that three different true models were used

for (d in c("COV0026")){# ', "COV0032", "COV0045"'

#-define coefficient for true covariate

true = ifelse(d == "COV0026", 0.026, ifelse(d == "COV0032", 0.032, 0.045)) coefficient

for (b in fremruns){

print(paste(d, "FREM data Cycle",b, Sys.time())) #live tracking for analysis via Rout data file

#-define patterns to extract the files of interest in the respective folder

pattern = paste(d,"/FREM/", b,"/frem_results.csv", sep="")

pattern2 = paste(d,"/FREM/", b,"/results.csv", sep="")

pattern3 = paste(d,"/FREM/", b,"/final_models/sdtab002", sep="")

####################################

# PSN created frem_results.csv #

####################################

#-no frem_results.csv file was created, frem has no output

if(file.exists(pattern) == FALSE){

print(paste(pattern, "does not exist"))

frem = data.frame( est= NA,

PARAMETER = NA,

RUN = b,

IDs = NA,

CORR = NA,

SIM = NA,

DIR = d,

true = NA)

rep_list_coeff[[which(fremruns == b)]] = frem

}

#frem_results.csv was created, extract the information

if(file.exists(pattern) == TRUE){

frem_results = as.data.table(read.csv(paste(d,"/FREM/",b,"/frem_results.csv", sep = ""),

header = FALSE, stringsAsFactors = F))

#-extract table of interest via line numbers

index1 = which(frem_results$V1 == "FREM parameter-covariate coefficients" ) + 1

index2 = which(frem_results$V1 == "FREM parameter (unexplained) variability") - 1

frem = frem_results[index1: index2, ] #select only the part of interest

frem[frem == ''] = NA

#-create a data.frame

frem.extract = frem %>%

remove_empty("cols") %>%

row_to_names(row_number = 1) %>%

mutate("V3" = NULL) %>%

setnames(c("CONDITION", "CLCOV1", "CLCOV2", "CLCOV3"))

frem.extract = frem.extract[2, ] # select “each” coefficients

#-check filtered data in Rout file

print(frem.extract)

frem.extract = frem.extract %>%

pivot_longer(cols = starts_with("CL"), values_to = "est", names_to = "PARAMETER") %>%

mutate("RUN" = b) %>%

mutate(est = as.numeric(est)) %>%

as.data.frame() %>%

dplyr::select(est,CONDITION, PARAMETER, RUN )

#-separate folder name into information about scenario

matches <- regmatches(frem$RUN, gregexpr("[[:digit:]]+", frem$RUN))

runID = as.numeric(unlist(matches))[1:3]

#-add to new columns

frem$IDs = runID[1]

frem$CORR = runID[2]

frem$SIM = runID[3]

frem$DIR = d

#-add true covariate coefficient for rbias an rrmse calculations

frem$true = ifelse(frem$PARAMETER == "CLCOV1", true, 0)

rep_list_coeff[[which(fremruns == b)]] = frem

}

####################################

# PSN created results.csv #

####################################

#results.csv was not created, no output of frem

if(file.exists(pattern2) == FALSE){

print(paste(pattern2, "does not exsist"))

frem_cov = data.frame( parameter = NA,

covariate = NA,

condition = NA,

p5 = NA,

mean = NA,

p95 = NA,

RUN = b,

IDs = NA,

CORR = NA,

SIM = NA,

DIR = d,

SIGCOV = 0,

INFO = "FAILED" )

rep_list_cov[[which(fremruns == b)]] = frem_cov

}

#-results.csv was created and information is extracted

if(file.exists(pattern2) == TRUE){

frem_cov_results = as.data.table(read.csv(paste(d,"/FREM/", b,"/results.csv", sep = ""), header = FALSE,

stringsAsFactors = F))

#-extract table of interest

index3 = which(frem_cov_results$V1 == "covariate_effects")+1

index4 = which(frem_cov_results$V1 == "individual_effects")-1

frem_cov = filter(frem_cov_results[index3: index4, ])

frem_cov = frem_cov %>%

mutate_all(funs(na_if(., ""))) %>%

remove_empty("cols") %>%

row_to_names(row_number = 1) %>%

mutate("RUN" = b)

#-use folder name to add scenario information to dataset

matches.cov <- regmatches(frem_cov$RUN, gregexpr("[[:digit:]]+", frem_cov$RUN))

runID.cov = as.numeric(unlist(matches.cov))[1:3]

frem_cov$IDs = runID.cov[1]

frem_cov$CORR = runID.cov[2]

frem_cov$SIM = runID.cov[3]

frem_cov$DIR = d

#-Check for percentile overlapping with 1 (no effect on clearance) to identify which covariate was significant

in frem run

covariates= c("COV1", "COV2", "COV3")

for(c in covariates){

#-Note: The effect at the 5th percentile covariate value can be smaller or higher than the effect at the 95th

percentile

#-covariate value. Based on that, significance is flagged by non-overlapping confidence

#intervals at either the upper or lower end of the uncertainty band.

if(( frem_cov$mean[frem_cov$covariate == c & frem_cov$condition == "5th"] <

frem_cov$mean[frem_cov$covariate == c & frem_cov$condition == "95th"]) == TRUE){

frem_cov$SIGCOV[frem_cov$covariate == c] =

ifelse(frem_cov$p95[frem_cov$covariate == c & frem_cov$condition == "5th"] < 1 , 1,

ifelse(frem_cov$p5[frem_cov$covariate == c & frem_cov$condition == "95th"] > 1, 1,0))

}

if(( frem_cov$mean[frem_cov$covariate == c & frem_cov$condition == "5th"] >

frem_cov$mean[frem_cov$covariate == c & frem_cov$condition == "95th"]) == TRUE){

frem_cov$SIGCOV[frem_cov$covariate == c] =

ifelse(frem_cov$p5[frem_cov$covariate == c & frem_cov$condition == "5th"] > 1 , 1,

ifelse(frem_cov$p95[frem_cov$covariate == c &frem_cov$condition == "95th"] < 1, 1,0))}

}

#-identify how many covariates were significant per frem run. Note: We define a significant covariate only in

#cases where the effect at the 5th and 95th percentile of covariate value was not overlapping with 1.

#-2 covariates significant

if(length(frem_cov$SIGCOV[frem_cov$SIGCOV == 0]) == 2 & # 4x SIGCOV == 1

length(unique(frem_cov$covariate[frem_cov$SIGCOV == 1])) == 2){

frem_cov$INFO = "2 covariates significant"

}

if(length(frem_cov$SIGCOV[frem_cov$SIGCOV == 0]) == 1 &

length(unique(frem_cov$covariate[frem_cov$SIGCOV == 1])) == 3){

frem_cov$INFO = "2 covariates significant"

}

#-all covariates significant

if(length(frem_cov$SIGCOV[frem_cov$SIGCOV == 0]) == 0 ){

frem_cov$INFO = "all covariates significant"

}

#-no covariate significant

if (length(frem_cov$SIGCOV[frem_cov$SIGCOV == 0]) == 6 |

length(frem_cov$SIGCOV[frem_cov$SIGCOV == 0]) == 5) {

frem_cov$INFO = "no significant covariate"

}

if (length(frem_cov$SIGCOV[frem_cov$SIGCOV == 0]) == 4 &

length(unique(frem_cov$covariate[frem_cov$SIGCOV == 1])) > 1) {

frem_cov$INFO = "no significant covariate" }

if (length(frem_cov$SIGCOV[frem_cov$SIGCOV == 0]) == 3 &

length(unique(frem_cov$covariate[frem_cov$SIGCOV == 1])) ==3 ) {

frem_cov$INFO = "no significant covariate"

}

#-one covariate significant

if(length(frem_cov$SIGCOV[frem_cov$SIGCOV == 0]) >= 3 &

length(frem_cov$SIGCOV[frem_cov$SIGCOV == 0]) <= 4 &

length(unique(frem_cov$covariate[frem_cov$SIGCOV == 1])) == 1 ){

frem_cov$INFO = "1 covariate significant"

}

if (length(frem_cov$SIGCOV[frem_cov$SIGCOV == 0]) == 2 &

length(unique(frem_cov$covariate[frem_cov$SIGCOV == 1])) > 2) {

frem_cov$INFO = "1 covariate significant"

}

if (length(frem_cov$SIGCOV[frem_cov$SIGCOV == 0]) == 3 &

length(unique(frem_cov$covariate[frem_cov$SIGCOV == 1])) == 2 ) {

frem_cov$INFO = "1 covariate significant"

}

if (length(frem_cov$SIGCOV[frem_cov$SIGCOV == 0]) == 4 &

length(unique(frem_cov$covariate[frem_cov$SIGCOV == 1])) == 1) {

frem_cov$INFO = "1 covariate significant"

}

#-flag the runs, that identified only COV1 (true) with significant effect on clearance

frem_cov$COV1SIG[frem_cov$SIGCOV == 1 & frem_cov$covariate == "COV1" &

frem_cov$INFO == "1 covariate significant"] = 1

#-calculate total covariate effect on clearance

effect.diff = frem_cov %>%

mutate(mean = as.numeric(mean) ) %>%

group_by(covariate) %>%

summarise_at(vars(mean), diff) %>%

pivot_wider(names_from = covariate , values_from = c(mean))

frem_cov$COV1effect = effect.diff$COV1

frem_cov$COV2effect = effect.diff$COV2

frem_cov$COV3effect = effect.diff$COV3

#-flag which covariate had the biggest effect, FLAG them with 1

frem_cov = frem_cov %>%

mutate("cov1_highest" = ifelse(COV1effect > COV2effect & COV1effect > COV3effect, 1, 0)) %>%

mutate("cov2_highest" = ifelse(COV2effect > COV1effect & COV2effect > COV3effect, 1, 0)) %>%

mutate("cov3_highest" = ifelse(COV3effect > COV2effect & COV3effect > COV1effect, 1, 0))

rep_list_cov[[which(fremruns == b)]] = frem_cov

}

if(file.exists(pattern3) == TRUE){ #sdtab is present (created with model_4)

#-use .lst file to select population PK values in final frem model (by default called model_4)

# for each frem run

lst_frem = read.lst(paste(d, "/FREM/", b,"/final_models/model_4.lst", sep= ""))

frem_theta = data.frame(unlist(lst_frem["thetas"]))

frem_theta = frem_theta %>%

mutate("NUM" = seq(1:length(frem_theta$'unlist.lst_frem..thetas...'))) %>%

mutate("PARAMETER" = c("CL", "V", "PROP", "ADD", "CLCOV1", "CLCOV2", "CLCOV3")) %>%

#-Number 1- 3 are PK parameters, number 4 is the fixed additive error, higher numbers are related to

covariates

filter(NUM<4) %>%

mutate("RUN" = b) %>%

mutate("NUM" = NULL) %>%

rename("est" = "unlist.lst_frem..thetas...")

#-extract information from folder name about simulation scenario

matches.theta <- regmatches(frem_theta$RUN, gregexpr("[[:digit:]]+", frem_theta$RUN))

runID.cov = as.numeric(unlist(matches.theta))[1:3]

frem_theta$IDs = runID.cov[1]

frem_theta$CORR = runID.cov[2]

frem_theta$SIM = runID.cov[3]

frem_theta$DIR = d

frem_theta$true = ifelse(frem_theta$PARAMETER == "CL", 18,

ifelse(frem_theta$PARAMETER == "V", 400,

ifelse(frem_theta$PARAMETER == "PROP", 0.15,-99)))

frem_theta =frem_theta %>%

group_by(RUN,DIR) %>%

mutate(RUNDIR=paste(RUN, DIR))

rep_list_pop[[which(fremruns == b)]] <- frem_theta

}

}

#-collect the information for each covariate effect directory

rep_list_coeff.loop[[d]] <- bind_rows(rep_list_coeff)

rep_list_cov.loop[[d]] = bind_rows(rep_list_cov)

rep_list_pop.loop[[d]] <- bind_rows(rep_list_pop)

}

#-estimated frem coefficients

data_out_frem = dplyr::bind_rows(rep_list_coeff.loop)

data_out_frem_cov = dplyr::bind_rows(rep_list_cov.loop)

data_out_frem_cov$parameter = NULL

data_out_frem= data_out_frem %>%

group_by(RUN, DIR) %>%

mutate(RUNDIR=paste(RUN, DIR))

data_out_frem_cov = data_out_frem_cov %>%

group_by(RUN,DIR) %>%

mutate(RUNDIR=paste(RUN, DIR))

data_out_frem_cov$COV1SIG[is.na(data_out_frem_cov$COV1SIG)] = 0

#-set same names, as in scm

data_out_frem_cov$covariate[data_out_frem_cov$covariate == "COV1"] = "CLCOV1"

data_out_frem_cov$covariate[data_out_frem_cov$covariate == "COV2"] = "CLCOV2"

data_out_frem_cov$covariate[data_out_frem_cov$covariate == "COV3"] = "CLCOV3"

names(data_out_frem_cov)[1] = "PARAMETER"

data_out = left_join(data_out_frem_cov, data_out_frem, by = c("RUN","IDs","CORR","SIM","DIR" , "RUNDIR", "PARAMETER"))

#save frem run data

write.csv(data_out, file= "FREM.csv", row.names = F)

data_out_frem_pop1 = dplyr::bind_rows(rep_list_pop.loop)

data_out_frem_pop = dplyr::bind_rows(data_out_frem_pop1, data_out_frem)

write.csv(data_out_frem_pop, file="FREM_PK_parameter.csv", row.names = F)

1. **R code for ‘scm’ results extraction with forward inclusion backward elimination models.**

rm(list=ls())

library(data.table)

library(tidyverse)

library(zoo)

library(boot)

library(xpose4)

library(stringr)

library(MASS)

library(janitor)

library(stringr)

#- IDs of simulation study

IDs = c(20,50,100,500)

#-define correlations tested in the study

CORR = c(0,0.15,0.5,0.8,0.9)

#-define number of simulations

SIM = seq(1,1000)

#-name lists to collect folder names for each scenario

loop1 = list()

loop2 = list()

loop2a= list()

#-get folder names for each scenario

for( i in IDs){

for(c in CORR){

for(s in SIM){

loop1[[s]] = paste("SCM_RUN_",i, "IDs_", c*100, "corr_sim", s, sep ="")

}

loop2[[which(CORR == c)]]= as.character(unlist(loop1))

}

loop2a[[i]] =as.character(unlist(loop2))

}

scmruns = unique(as.character(unlist(loop2a)))

#-define names of lists for the analysis loop

rep_list_scm_tab_pop.loop = list()

rep_list_scm_tab_pop = list()

vector.is.empty <- function(x) return(length(x) ==0 )

for (d in c("COV0026", "COV0032" and "COV0045" )){

true = ifelse(d == "COV0026", 0.026, ifelse(d == "COV0032", 0.032, 0.045))

for(l in scmruns){

print(l)

pattern4 = paste( d,"/SCM/", l,"/final_models/final_backward.lst",sep="")

pattern5 = paste( d,"/SCM/", l,"/final_models/final_forward.lst" ,sep="")

#-define pattens to search for in mod file

cov_pattern =";;; CLCOV"

FILE_pattern = "FILE=/home/YOUR_DIRECTORY/"

cov_def = ") ; CLCOV"

cov1 = "CLCOV11"

cov2 = "CLCOV21"

cov3 = "CLCOV31"

######################################

# NO COVARIATE FOUND #

######################################

if(file.exists(pattern4) == FALSE & file.exists(pattern5) == FALSE) {

matches <- regmatches(l, gregexpr("[[:digit:]]+", l))

runID = as.numeric(unlist(matches))[1:3]

mod.rerun = scan(paste("run001_est.mod",sep=""), sep = "\n", what = character(), quiet = TRUE)

#-run model with COV1 relationship included to obtain a coefficient for the “all to all” comparison

#-navigate to dataset used for scm

mod.rerun[19] = paste("$DATA cov_temp", runID[1], "IDs", runID[2], "corr_sim", runID[3] ,".csv

IGNORE=@",sep = "")

#-adjust initial estimate to simulated covariate effect size

if(d == "COV0026"){ mod.rerun[46] = "(0.026) ; 5_CL-COV1" }

if(d == "COV0032"){ mod.rerun[46] = "(0.032) ; 5_CL-COV1" }

if(d == "COV0045"){ mod.rerun[46] = "(0.045) ; 5_CL-COV1" }

#-write modfile

write(mod.rerun, paste( d, "/SCM/" ,l,"/run001_est.mod",sep=""))

#-execute runs

system(paste("execute -model_dir ", d, "/SCM/" ,l,"/run001_est.mod", " -clean=3 -silent",sep=""), wait = T ,

intern = F)

#-read thetas in .lst file

lst_scm_rerun = read.lst(paste( d, "/SCM/", l,"/run001_est.lst", sep= ""))["thetas"]

#-extract estimated coefficient of true covariate

scm_theta = data.frame(unlist(lst_scm_rerun["thetas"]))

scm_pop = scm_theta %>%

mutate("NUM" = seq(1:length(scm_theta$unlist.lst_scm_rerun..thetas...))) %>%

mutate("PARAMETER" = c("CL", "V", "PROP", "ADD", "CLCOV11")) %>%

mutate("RUN" = l) %>%

mutate("NUM" = NULL) %>%

rename("est" = "unlist.lst_scm_rerun..thetas...")

scm_pop$SIG = 0

scm_pop$model = "no covariate selected"

scm_pop$IDs = runID[1]

scm_pop$CORR = runID[2]

scm_pop$SIM = runID[3]

scm_pop$DIR = d

scm_pop$true[scm_pop$PARAMETER == "CL"] = 18

scm_pop$true[scm_pop$PARAMETER == "V"] = 400

scm_pop$true[scm_pop$PARAMETER == "PROP"] = 0.15

scm_pop$true[scm_pop$PARAMETER == "CLCOV11"] = ifelse(d == "COV0026", 0.026,

ifelse(d == "COV0032", 0.032, 0.045))

#-assure numeric columns

scm_pop$est = as.numeric(scm_pop$est)

scm_pop$IDs = as.numeric(scm_pop$IDs)

scm_pop$CORR = as.numeric(scm_pop$CORR)

scm_pop$SIM = as.numeric(scm_pop$SIM)

scm_pop$true= as.numeric(scm_pop$true)

scm_pop$SIG= as.numeric(scm_pop$SIG)

scm_pop$METHOD = "SCM"

scm_pop$RUNDIR = paste(scm_pop$RUN, scm_pop$DIR)

rep_list_scm_tab_pop[[which(scmruns == l)]] <- scm_pop

}#-close “no covariate found”

###############################################

# BACKWARD MODEL PRESENT #

# EITHER WITH OR WITHOUT COVARIATE #

###############################################

if(file.exists(pattern4) == TRUE){ #check if final backward file is present

print("final backward model present")

mod = scan(paste( d,"/SCM/", l,"/final_models/final_backward.mod",sep=""), sep = "\n", what = character(),

quiet = TRUE)

#-search for covariate relationship

search_cov_rel = grep(cov_pattern, mod)

matches <- regmatches(l, gregexpr("[[:digit:]]+", l))

runID = as.numeric(unlist(matches))[1:3]

###########################################

# BACKWARD MODEL PRESENT #

# WITHOUT COVARIATE #

###########################################

#-empty backward model

if(vector.is.empty(search_cov_rel) == TRUE){

mod.rerun= scan(paste("run001_est.mod",sep=""), sep = "\n", what = character(), quiet = TRUE)

#-navigate to simulated dataset, which was used in the scm run

mod.rerun[19] = paste("$DATA cov_temp", runID[1], "IDs", runID[2], "corr_sim", runID[3] ,".csv

IGNORE=@", sep = "")

if(d == "COV0026"){ mod.rerun[46] = "(0.026) ; 5_CL-COV1" }

if(d == "COV0032"){ mod.rerun[46] = "(0.032) ; 5_CL-COV1" }

if(d == "COV0045"){ mod.rerun[46] = "(0.045) ; 5_CL-COV1" }

#-write modfile

write(mod.rerun, paste( d, "/SCM/" ,l,"/run001_est.mod",sep=""))

#-execute run

system(paste("execute -model_dir ", d, "/SCM/" ,l,"/run001_est.mod", " -clean=3 -silent",sep=""),

wait = T , intern = F)

#-read thetas in lst file

lst_scm_rerun = read.lst(paste(d, "/SCM/", l,"/run001_est.lst", sep= ""))["thetas"]

scm_theta = data.frame(unlist(lst_scm_rerun["thetas"]))

scm_pop = scm_theta %>%

mutate("NUM" = seq(1:length(scm_theta$unlist.lst_scm_rerun..thetas...))) %>%

#-use the name of the cov relation

mutate("PARAMETER" = c("CL", "V", "PROP", "ADD", "CLCOV11")) %>%

mutate("RUN" = l) %>%

mutate("NUM" = NULL) %>%

rename("est" = "unlist.lst_scm_rerun..thetas...")

scm_pop$model = "backward_NOCOV"

scm_pop$SIG = 0

} #close “final backward model without covariate”

###################################################

# BACKWARD MODEL WITH COVARIATE #

###################################################

if(vector.is.empty(search_cov_rel) == FALSE) { #if vector includes a number, a covariate was selected

mod_scm = gsub("IPRED IWRES CWRES", as.character("COVCOEFF IPRED IWRES CWRES"), mod,

ignore.case=T)

mod_scm2 = gsub(";FREELINE1;", as.character("COVCOEFF = THETA(5)"),mod_scm, ignore.case=T)

file_repl = grep(FILE_pattern, mod_scm2)

#pop-PK data in one table per scm run

lst_scm = read.lst(paste( d,"/SCM/", l,"/final_models/final_backward.lst", sep= ""))

scm_theta = data.frame(unlist(lst_scm["thetas"]))

search_scm = grep(cov_def, mod_scm2) # need to know which covariate was selected

mod_search_scm = mod_scm2[search_scm]

scm_pop = scm_theta %>%

mutate("NUM" = seq(1:length(scm_theta$'unlist.lst_scm..thetas...'))) %>%

#-ass PK parameter names and use the name of the selected covariate

mutate("PARAMETER" = c("CL", "V", "PROP", "ADD", word(mod_search_scm, -1))) %>%

filter(NUM!=4) %>% # num. 4 is add error, which is fixed

mutate("RUN" = l) %>%

mutate("NUM" = NULL) %>%

rename("est" = "unlist.lst_scm..thetas...")

scm_pop$model = "backward"

scm_pop$SIG = 1

#########################################################

# BACKWARD MODEL WITH WRONG COVARIATE #

#########################################################

if(scm_pop[4,2] %in% c("CLCOV21", "CLCOV31")){

matches <- regmatches(scm_pop$RUN, gregexpr("[[:digit:]]+", scm_pop$RUN))

runID = as.numeric(unlist(matches))[1:3]

#-read in model file with $ESTIMATION

mod.rerun = scan(paste("run001_est.mod",sep=""), sep = "\n", what = character(),

quiet = TRUE)

#-use simulated dataset, which was used in the scm run

mod.rerun[19] = paste("$DATA cov_temp", runID[1], "IDs",

runID[2], "corr_sim", runID[3] ,".csv IGNORE=@",

sep = "")

if(d == "COV0026"){ mod.rerun[46] = "(0.026) ; 5_CL-COV1" }

if(d == "COV0032"){ mod.rerun[46] = "(0.032) ; 5_CL-COV1" }

if(d == "COV0045"){ mod.rerun[46] = "(0.045) ; 5_CL-COV1" }

#-write modfile

write(mod.rerun, paste( d, "/SCM/" ,l,"/run001_est.mod", sep="" ))

#-execute runs

system(paste("execute -model_dir ", d, "/SCM/",l, "/run001_est.mod -clean=3 -silent",sep=""),

wait = T , intern = F)

#-read thetas out of .lst file

lst_scm_rerun = read.lst(paste( d,"/", l,"/run001_est.lst", sep= ""))["thetas"]

#-extract estimated coefficient of true covariate

coeff.cov1 = unlist(lst_scm_rerun)[5]

#add row to scm_pop data frame with estimate, parameter name, folder name, non-significance

Information

#-add coefficient for true covariate

scm_pop[nrow(scm_pop)+1, ] = c(coeff.cov1, "CLCOV1", l, 0, "backward_wrong cov")

}

} # close “final backward model with covariate”

#-add additional information in scm_pop data.frame

scm_pop$IDs = runID[1]

scm_pop$CORR = runID[2]

scm_pop$SIM = runID[3]

scm_pop$DIR = d

scm_pop$true[scm_pop$PARAMETER == "CL"] = 18

scm_pop$true[scm_pop$PARAMETER == "V"] = 400

scm_pop$true[scm_pop$PARAMETER == "PROP"] = 0.15

scm_pop$true[scm_pop$PARAMETER == "CLCOV11"] = ifelse(d == "COV0026", 0.026,

ifelse(d == "COV0032", 0.032, 0.045))

scm_pop$true[scm_pop$PARAMETER == "CLCOV21"] = 0

scm_pop$true[scm_pop$PARAMETER == "CLCOV31"] = 0

scm_pop$est = as.numeric(scm_pop$est)

scm_pop$IDs = as.numeric(scm_pop$IDs)

scm_pop$CORR = as.numeric(scm_pop$CORR)

scm_pop$SIM = as.numeric(scm_pop$SIM)

scm_pop$true= as.numeric(scm_pop$true)

scm_pop$SIG= as.numeric(scm_pop$SIG)

scm_pop$METHOD = "SCM"

scm_pop$RUNDIR = paste(scm_pop$RUN, scm_pop$DIR)

rep_list_scm_tab_pop[[which(scmruns == l)]] <- scm_pop

} #close “final backward model”

############################################

# FORWARD MODEL PRESENT #

############################################

#-check if final forward file is present, use the forward model only if this is the final model (no backward

#model present)

if(file.exists(pattern5) == TRUE & file.exists(pattern4) == FALSE){

mod = scan(paste( d,"/SCM/", l,"/final_models/final_forward.mod",sep=""), sep = "\n", what = character(),

quiet = TRUE)

#-search for covariate relationship

search_cov_rel = grep(cov_pattern, mod)

matches <- regmatches(l, gregexpr("[[:digit:]]+", l))

runID = as.numeric(unlist(matches))[1:3]

#########################################

# FORWARD MODEL, NO COVARIATE #

#########################################

if(vector.is.empty(search_cov_rel) == TRUE) {

mod.rerun= scan(paste("run001_est.mod",sep=""), sep = "\n", what = character(), quiet = TRUE)

#-navigate to simulated dataset, which was used in the scm run

mod.rerun[19] = paste("$DATA cov_temp", runID[1], "IDs", runID[2], "corr_sim", runID[3] ,".csv

IGNORE=@", sep = "")

if(d == "COV0026"){ mod.rerun[46] = "(0.026) ; 5_CL-COV1" }

if(d == "COV0032"){ mod.rerun[46] = "(0.032) ; 5_CL-COV1" }

if(d == "COV0045"){ mod.rerun[46] = "(0.045) ; 5_CL-COV1" }

#-write modfile

write(mod.rerun, paste( d, "/SCM/" ,l,"/run001_est.mod",sep=""))

#-execute run

system(paste("execute -model_dir ", d, "/SCM/" ,l,"/run001_est.mod", " -clean=3 -silent",sep=""),

wait = T , intern = F)

#-read thetas in lst file

lst_scm_rerun = read.lst(paste(d, "/SCM/", l,"/run001_est.lst", sep= ""))["thetas"]

#-extract estimates coefficient of true covariate

coeff.cov1 = unlist(lst_scm_rerun)[5]

#add row to scm_pop data frame with estimate, parameter name, folder name, non-significance information

scm_theta = data.frame(unlist(lst_scm_rerun["thetas"]))

scm_pop = scm_theta %>%

mutate("NUM" = seq(1:length(scm_theta$unlist.lst_scm_rerun..thetas...))) %>%

#-use the name of the cov relation

mutate("PARAMETER" = c("CL", "V", "PROP", "ADD", "CLCOV1")) %>%

mutate("RUN" = l) %>%

mutate("NUM" = NULL) %>%

rename("est" = "unlist.lst_scm_rerun..thetas...")

scm_pop$SIG = 0

scm_pop$model = "forward_NOCOV"

} #close “final forward, no covariate”

############################################

# FORWARD MODEL WITH COVARIATE #

############################################

if(vector.is.empty(search_cov_rel) == FALSE) { #if vector includes a number, a covariate was selected

#-pop-PK data per scm run

lst_scm = read.lst(paste( d,"/SCM/", l,"/final_models/final_forward.lst", sep= ""))

scm_theta = data.frame(unlist(lst_scm["thetas"]))

search_scm = grep(cov_def, mod) # need to know the position

mod_search_scm = mod[search_scm]

scm_pop = scm_theta %>%

mutate("NUM" = seq(1:length(scm_theta$'unlist.lst_scm..thetas...'))) %>%

#-use the name of the cov relation

mutate("PARAMETER" = c("CL", "V", "PROP", "ADD", word(mod_search_scm, -1))) %>%

filter(NUM!=4) %>% # num. 4 is add error, which is fixed, higher numbers are covariate related

mutate("RUN" = l) %>%

mutate("NUM" = NULL) %>%

rename("est" = "unlist.lst_scm..thetas...")

scm_pop$SIG = 1

scm_pop$model = "forward"

#-In cases a wrong covariate (COV2 or COV3) was included in the final model, use the model with

#-covariate relationship to obtain a COV1 coefficient for the ‘all to all’ comparison.

####################################################

# FORWARD MODEL WITH WRONG COVARIATE #

####################################################

if(scm_pop[4,2] %in% c("CLCOV21", "CLCOV31")){

#-read in model file with $ESTIMATION (true model with $EST instead of $SIM)

mod.rerun = scan(paste("run001_est.mod",sep=""), sep = "\n", what = character(),

quiet = TRUE)

#-navigate to simulated dataset, which was used in the scm run

mod.rerun[19] = paste("$DATA cov_temp", runID[1], "IDs", runID[2], "corr_sim",

runID[3] ,".csv IGNORE=@",

sep = "")

#-set the initial estimate to the true value according to the d

if(d == "COV0026"){ mod.rerun[46] = "(0.026) ; 5_CL-COV1" }

if(d == "COV0032"){ mod.rerun[46] = "(0.032) ; 5_CL-COV1" }

if(d == "COV0045"){ mod.rerun[46] = "(0.045) ; 5_CL-COV1" }

#-write modfile

write(mod.rerun, paste(d, "/SCM/",l,"/run001_est.mod",sep=""))

#-execute runs

system(paste("execute -model_dir ", d , "/SCM/", l,"/run001_est.mod -clean=3",sep=""),

wait = T , intern = F)

#-read thetas in .lst file

lst_scm_rerun = read.lst(paste(d,"/SCM/", l,"/run001_est.lst", sep= ""))["thetas"]

#-extract coefficient of true covariate

coeff.cov1 = unlist(lst_scm_rerun)[5]

#-add row to scm_pop data frame with estimate, parameter name, folder name, non-significance information

scm_pop[nrow(scm_pop)+1, ] = c(coeff.cov1, "CLCOV1", l, 0, "forward_wrong_cov")

} #close “final forward with wrong covariate”

}#close “final forward with covariate”

matches <- regmatches(scm_pop$RUN, gregexpr("[[:digit:]]+", scm_pop$RUN))

runID = as.numeric(unlist(matches))[1:3]

scm_pop$IDs = runID[1]

scm_pop$CORR = runID[2]

scm_pop$SIM = runID[3]

scm_pop$DIR = d

scm_pop$true[scm_pop$PARAMETER == "CL"] = 18

scm_pop$true[scm_pop$PARAMETER == "V"] = 400

scm_pop$true[scm_pop$PARAMETER == "PROP"] = 0.15

scm_pop$true[scm_pop$PARAMETER == "CLCOV11"] = ifelse(d == "COV0026", 0.026,

ifelse(d == "COV0032", 0.032, 0.045))

scm_pop$true[scm_pop$PARAMETER == "CLCOV1"] = ifelse(d == "COV0026", 0.026,

ifelse(d == "COV0032", 0.032, 0.045))

scm_pop$true[scm_pop$PARAMETER == "CLCOV21" | scm_pop$PARAMETER == "CLCOV31" ] = 0

scm_pop$est = as.numeric(scm_pop$est)

scm_pop$IDs = as.numeric(scm_pop$IDs)

scm_pop$CORR = as.numeric(scm_pop$CORR)

scm_pop$SIM = as.numeric(scm_pop$SIM)

scm_pop$true= as.numeric(scm_pop$true)

scm_pop$SIG= as.numeric(scm_pop$SIG)

scm_pop$METHOD = "SCM"

scm_pop$RUNDIR = paste(scm_pop$RUN, scm_pop$DIR)

rep_list_scm_tab_pop[[which(scmruns == l)]] <- scm_pop

} #close “final forward model”

} #close l loop

rep_list_scm_tab_pop.loop[[d]] = bind_rows(rep_list_scm_tab_pop)

} # close d loop

scm_pop_out = bind_rows(rep_list_scm_tab_pop.loop)

scm_pop_out$PARAMETER[scm_pop_out$PARAMETER == "CLCOV11"] = "CLCOV1"

scm_pop_out$PARAMETER[scm_pop_out$PARAMETER == "CLCOV21"] = "CLCOV2"

scm_pop_out$PARAMETER[scm_pop_out$PARAMETER == "CLCOV31"] = "CLCOV3"

scm_pop_out$PARAMETER = as.character(scm_pop_out$PARAMETER)

scm_pop_out$RUN = as.character(scm_pop_out$RUN)

scm_pop_out$model = as.character(scm_pop_out$model)

scm_pop_out$true[scm_pop_out$PARAMETER == "ADD"] = 0.001

write.csv(scm_pop_out, file= paste("SCM_DATA ",Sys.Date(), sep =""),

row.names = F)
