# Supplementary material for: Operational characteristics of full random effects modelling (‘frem’) compared to stepwise covariate modelling (‘scm’)
Source: J Pharmacokinet Pharmacodyn. 2023 Apr 21;50(4):315–26. doi: 10.1007/s10928-023-09856-w (PMC10374720; doi:10.1007/s10928-023-09856-w)
Supplement: Supplementary file 2 — Supplementary file2 (DOCX 1059 kb) [file 10928_2023_9856_MOESM2_ESM.docx]

**Supplement 2**

**Simulation study using a true categorical covariate.**

In this simulation sub study, we investigated 0 % and 80 % correlation between the true dichotomous categorical covariate and covariate_II_. Covariate_III_ was independent of the other two covariates and represents pure noise. Covariate values were sampled with the code below.

#--------------------------------------------------------------------------------------------------------------

#-define number of samples

N <-500

#-set the means

mu <- c(28,8)

#-define correlation matrix between the two continuous covariates, here correlation 0 %

sigma <- matrix(c(15, 0, 0, 1.2 ),2,2)

#-set a seed number

set.seed(seed[a])

#-sample

df <- as.data.frame(mvrnorm(n=N, mu=mu, Sigma=sigma))

#set up the data frame with a categorical covariate correlated by 80 % to one of the continuous covariates

df1<-df%>%

mutate(CATEGORICAL = case_when(V1<quantile(V1,0.25) ~ sample(c(1,0), n(),

replace = TRUE, p = c(1,0)),

V1<quantile(V1,0.5) ~ sample(c(1,0), n(), replace = TRUE, p = c(1,0)),

V1>quantile(V1,0.5) ~ sample(c(1,0), n(), replace = TRUE, p = c(0,1)),

V1>quantile(V1,0.75) ~ sample(c(1,0), n(), replace = TRUE, p = c(0,1))))

#Check correlation

round(cor(df1)[3,1], digits= 3)

#-----------------------------------------------------------------------------------------------------------

All simulations used individually simulated datasets with 20, 50, 100, 500 virtual patients (n) including 2 (sparse) observations per individual. PK profiles of the scenarios (1-CMT PK model, i.v short infusion, linear elimination) were obtained via Monte Carlo simulations. The true model included the categorical covariate as fractional change on clearance (Eq. 1)

IF(CATEGORICAL.COV == 0) CL = THETA(1) (Eq. 1)

IF(CATEGORICAL.COV == 1) CL = THETA(1) * (1+θ_cat-cov_ )

Simulated coefficients represented a covariate effect of -20 % or -40 % (-0.2, -0.4) on clearance. In cases that a categorical covariate of 1 was identified as the ‘reference’ value, the true value is +0.25 or +0.67.

Beside interindividual variability on clearance (IIV_CL_: 0.1 variance, log-normal distribution), this sub study compared simulations in presence with and without inter individual variability on central volume of distribution (IIV_V_: 0.2 variance, log-normal distribution). PK parameter estimation was performed with first order conditional estimation with interaction (FOCE+I), using the simulated datasets (n = 1000) and a structural model without any covariates included. From here 500 ‘scm’ and ‘frem’ runs were executed. Scenario 1 simulated an comparison applying ‘scm’ forward selection (*p* < 0.05) and a backward elimination (*p* < 0.01), as well as applied only ‘scm’ forward selection (p< 0.1) as comparison to ‘frem_posthoc_’ (scenario 2). We evaluated the power to select/identify the true categorical covariate, but also conditional accuracy (Eq. 2) and precision (Eq. 3) of the estimates. For ‘frem_posthoc_’ the mean effect of ‘*other’* compared to ‘*reference’* served for calculation of the fractional change coefficient. Whether 0 or 1 was the reference in the datasets was extracted from the PSN provided ‘results.csv’ file.

$rBIAS \left[ \% \right]= \frac{1}{N}\cdot\sum_{1}^{i} \frac{{(estimated}_{i}- {true}_{i})}{{true}_{i}}\cdot100$ (Eq. 2)

$rRMSE \left[ \% \right]=\sqrt{\frac{1}{N}\cdot\sum_{1}^{i} \frac{{(estimated}_{i}-{true}_{i})^{2}}{{true}_{i}^{2}}} \cdot100$ (Eq. 3)

**Scenario 1 (categorical covariate)**

The results of scenario 1 are displayed in Figure S2- 3. For ‘frem_posthoc_’ models with a significant cov_true_ effect were evaluated. The power to identify the true categorical covariate was 47 % (‘frem_posthoc_’) vs. 28 % (‘scm’) in the scenario with n = 20, cov-corr 80 % and the covariate effect being -20 % on clearance. With increasing the covariate effect size to -40 % we revealed a power of 89 % using the ‘frem_posthoc_’ and 74 % in ‘scm’ (n = 20, cov-corr: 80 %). Thus, the observed behavior of power was similar as in the study handling continuous covariates. Power increased with increasing covariate effect size and was reduced in presence of 80 % cov-corr. In large datasets (n= 500), both methods approached 100 % power.

Additionally, we investigated the presented scenarios without inter individual variability on central volume of distribution (IIVV) to investigate its impact. The power of the ‘frem_posthoc_ method increased from e.g., from 89 % to 95 % (n = 20, cov-corr: 80 %, -40 % on clearance). For ‘scm’ power was increased in this scenario from 74 % to 78 %.

Besides that, the correlated continuous covariate (80 %) had a significant effect in ≥ 79 % of the ‘frem_posthoc_’ models and in ≥ 95 % in large datasets (n = 500). In contrast to that, ‘scm’ included the true categorical covariate together with covariate_II_ in none of the models. As a single covariate, ‘scm’ selected covariate_II_ at a maximum of 15 %.

Moreover, the independent, non-correlated covariate (covariate_III_) had a significant effect in 10 % (n=500) - 16 % (n = 20) of ‘frem’ runs and between 4 – 9 % in ‘scm’ runs.

Conditional accuracy and precision were functions of power. Rbias was strongly reduced with increasing power and the effect of correlation had only minor impact, regardless of the method. ‘Scm’ (-5.3 to 7 %) and ‘frem_posthoc_’ (-4 to 0.2 %) estimates were slightly biased in small n datasets, if the covariate effect was strong (-40 %). In the simulations without IIVV rbias was comparable (9 % to 12 %, covariate effect: -40 %).

The rrmse of the true covariate coefficient estimated, obtained via ‘scm’ was reduced from 110 % to 15 % (n = 20 – 500, - 20%) or with increasing effect size from 110 % to 43 % (n=20, 80 % correlation). Without IIVV, rrmse was reduced from 108 % to 13 % (‘scm’, n= 20-500, 80 % corr, -20 %) and from 108 % to 39 % with increasing effect size of the covariate (‘scm’, n= 20, 80 % corr, -20 %).

Across all simulated scenarios, 6/16000 ‘frem’ models provided an estimated true covariate effect of >2500 % on clearance, so that these models were excluded for the evaluations as these outliers would blur the statistics.

**
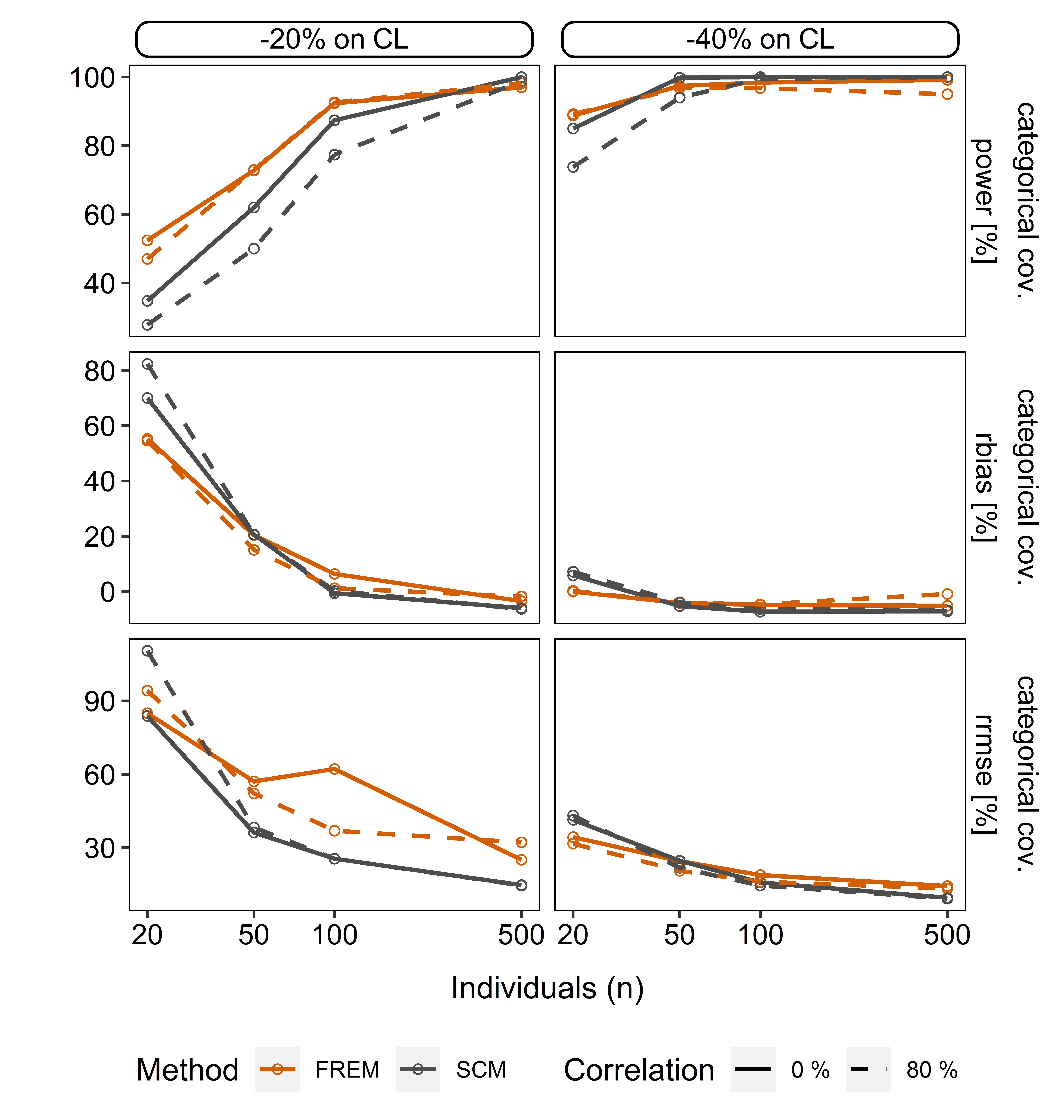
**

Figure S2- 3 Power precision and accuracy of the true categorical covariate (cat. cov) in the comparison of ‘scm’ (forward selection, p<0.05; backward elimination p>0.01) vs. ‘frem_posthoc_’ for two covariate effect sizes (scenario  1)

**Scenario 2 (categorical covariate)**

For this comparison the scm was performed with forward selection only (p<0.1), as a direct comparison to the 90 % confidence interval of the covariate effects in the ‘frem_posthoc_’ method.

The overall results followed the same trend as observed for the true continuous covariate but were also comparable to scenario 1 (Figure S2- 4). Power increased from 40 % to 100 % (‘scm’, n=20-500, cov-corr: 0 %, covariate effect: -20 %) and from 40 % to 89 % with increasing covariate effect size.

Rbias decreased from 57 %/48 % to -5.8 %/-1.4 % and rrmse from 94 %/84 % to 14 %/29 % (‘scm’/’frem’, n=20-500, cov-corr: 0 %, covariate effect: -20 %), as a function of power. In the scenarios with -40 % covariate effect size, power was strongly increased (> 74 %) and rbias was between 2.2 % and -7.4 %. The correlated continuous covariate_II_ was statistically significant in 39 % of simulated small n datasets (n=20, covariate correlation 80 %, covariate effect: ‑20 %) and in 95 % in large datasets (n=500). In contrast to that, none of the final forward ‘scm’ models included both covariates.

The independent covariate_III_ had a significant effect in 15 – 10 % (n = 20 – 500) of the final ‘frem’ models with a mean error between -0.017 - 0.02. The alpha value for ‘scm’ was between 6 and 12 %.

**
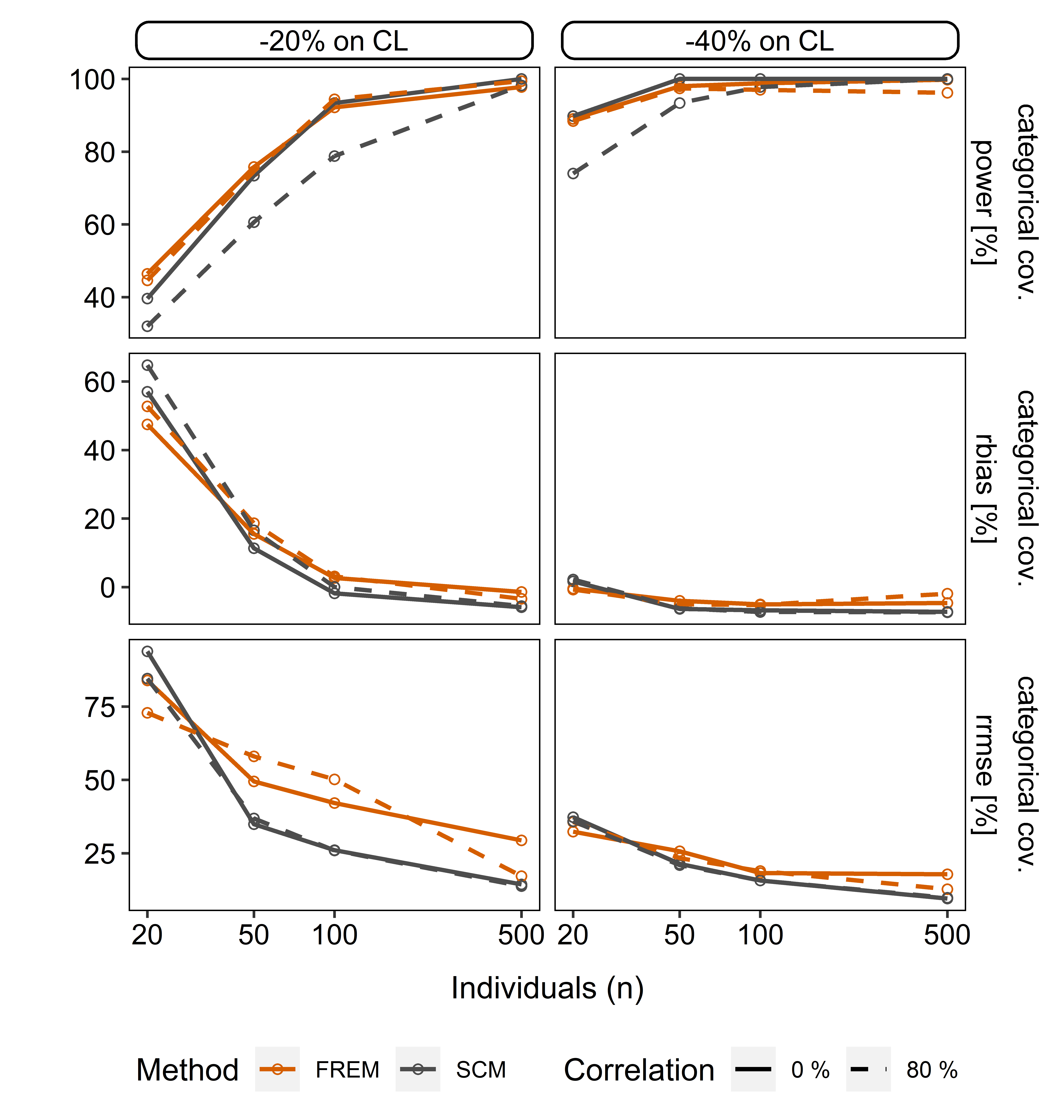
**

Figure S2- 4 Power, precision, and accuracy of the true categorical in scenario 2 of ‘scm’ (forward selection, p<0.1) and ‘fremposthoc’. The covariate effect on clearance was -20 %, -40 % respectively.
