# Supplementary material for: Operational characteristics of full random effects modelling (‘frem’) compared to stepwise covariate modelling (‘scm’)
Source: J Pharmacokinet Pharmacodyn. 2023 Apr 21;50(4):315–26. doi: 10.1007/s10928-023-09856-w (PMC10374720; doi:10.1007/s10928-023-09856-w)
Supplement: Supplementary file 3 — Supplementary file3 (DOCX 2671 kb) [file 10928_2023_9856_MOESM3_ESM.docx]

**Supplement 3**


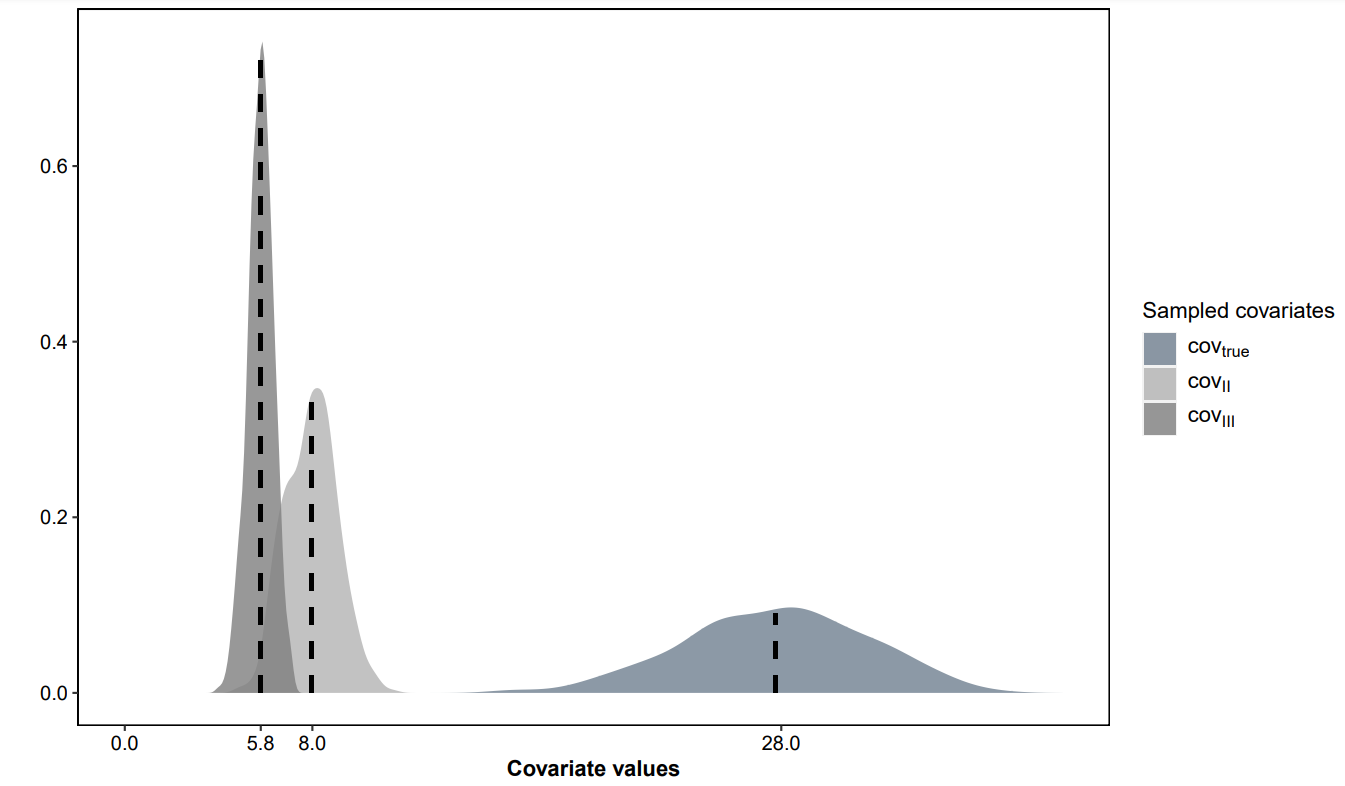


Figure S3 - 1 Multivariate normal distribution of continuous covariate values with the mean displayed as vertical lines

**Results for Scenario 1**





Figure S3 - 2 Density plot with estimated covariate coefficients for scenario 1 with three different covariate relative effect sizes on clearance (CL). The vertical line denotes the true covariate coefficient.

**

**Figure S3 - 3 Fraction of predictive performance of ‘scm’ and ‘frem_posthoc_’ (scenario 1, continuous covariates) against single components (a: N, b: relative root mean squared error (rrmse), c: relative bias (rbias), d: covariate effect size, e: covariate correlation) of this simulation study. Covariate coefficient estimates between zero and two times the true value were termed predictive

**Results for Scenario 2**

‘Frem_posthoc_’ results were compared to ‘scm’ forward inclusion models (p-value <0.1) to provide a statistically similar ‘head-to-head’ comparison. Throughout scenario 2, the ‘scm’ power to include cov_true_ was less affected by correlation compared to ‘frem_posthoc_’ (Figure 3 and Table S3 - 1). In small n datasets, the ‘scm’ performed slightly better with a maximum difference in power of +23 % (n = 20, $\theta_{{cov}_{true}} = 0.026$).

For ‘frem_posthoc_’, the frequency of significant cov_II_ inclusions in the final ‘frem_posthoc_’ models was > 74 % in presence of ≥ 80 % correlation between the covariates (n ≥ 100). In contrast to that, cov_II_ was significantly included in < 23 % of ‘scm’ runs with a maximum mean error (me) of cov_II_ estimate of 0.2 (n=20, $\theta_{{cov}_{true}}=0.045$, cov-corr 90 %). The highest mean error of ‘frem_posthoc_’ cov_II_ estimates was 0.18 (n=20, $\theta_{{cov}_{true}}=0.045$, cov-corr 90 %).

Overestimation of the $\theta_{{cov}_{true}}$ was observed for ‘frem_posthoc_’ and ‘scm’, favouring ‘scm’ in small n datasets. The rbias of frem_posthoc_ $\theta_{{cov}_{true}}$ coefficients were reduced with an increasing number of study subjects, but also with increasing relative covariate effect size (Table S3 - 1). In large datasets, estimated covariate coefficients were unbiased. Although power differences were observed, the fraction of predictive models in scenario 2 were similar (scm: 97.0 % ‘frem_posthoc_’:97.5 %, n = 50, cov-corr = 80 %, $\theta_{{cov}_{true}}=0.026$ ) and reached both 100 % in the scenario with the highest simulated covariate effect magnitude ($\theta_{{cov}_{true}}=0.045$, n > 50), see Figure S3 - 4. Conditional accuracy and precision for each method is presented in Table S3 - 1.

The direct comparison of the estimated ‘frem_posthoc_’ and ‘scm’ coefficients (based on the same dataset) is displayed in Figure S3 - 5. This representation of the results shows that ‘frem_posthoc_’ estimates are similar to ‘scm’ in case both methods found a significant cov_true_ relationship based on the same dataset.

Table S3 - 1 ‘Scm’ and ‘fremposthoc’ simulation results of scenario 2. The relative covariate effect sizes were -18 - +22 % ($\theta_{{cov}_{true}}$_true ) 0.026), -22 to +27 % ( $\theta_{{cov}_{true}}$0.032) and -29 to +41 % ($\theta_{{cov}_{true}}$ 0.045 ) on clearance.

| **N** | **Covariate correlation [%]** | **Method** | $\boldsymbol{\theta}_{\boldsymbol{cov}_{\boldsymbol{true}}}\mathbf{0}.\mathbf{026}$ | | | $\boldsymbol{\theta}_{\boldsymbol{cov}_{\boldsymbol{true}}}\mathbf{0.032}$ | | | | $\boldsymbol{\theta}_{\boldsymbol{cov}_{\boldsymbol{true}}}\mathbf{0.045}$ | | |
| --- | --- | --- | --- | --- | --- | --- | --- | --- | --- | --- | --- | --- |
|  |  |  | power  [%] | rbias [%] | rrmse [%] | power  [%] | rbias [%] | rrmse [%] | power  [%] | | rbias [%] | rrmse [%] |
| **20** | 0 | ‘frem’ | 40.7 | 69.3 | 88.4 | 50.1 | 43.1 | 64.7 | 71.1 | | 21.9 | 43.9 |
|  |  | ‘scm’ | 56.0 | 38.6 | 88.0 | 63.9 | 28.4 | 64.5 | 78.6 | | 16.7 | 44.1 |
|  | 50 | ‘frem’ | 40.0 | 70.3 | 88.5 | 45.1 | 43.5 | 64.7 | 65.5 | | 20.5 | 43.0 |
|  |  | ‘scm’ | 56.7 | 41.7 | 87.9 | 63.2 | 28.9 | 64.3 | 77.9 | | 14.8 | 43.5 |
|  | 90 | ‘frem’ | 29.9 | 67.3 | 86.9 | 33.2 | 44.1 | 66.6 | 46.7 | | 20.7 | 43.1 |
|  |  | ‘scm’ | 52.7 | 47.6 | 87.9 | 61.0 | 33.6 | 64.0 | 76.5 | | 16.5 | 42.7 |
| **50** | 0 | ‘frem’ | 67.0 | 26.5 | 42.7 | 80.1 | 14.4 | 34.4 | 95.5 | | 3.40 | 26.2 |
|  |  | ‘scm’ | 77.1 | 17.5 | 42.2 | 86.2 | 9.5 | 35.1 | 97.3 | | 1.70 | 26.7 |
|  | 50 | ‘frem’ | 62.6 | 26.5 | 43.1 | 75.4 | 15.3 | 34.9 | 91.7 | | 2.30 | 25.9 |
|  |  | ‘scm’ | 76.1 | 18.0 | 41.9 | 86.2 | 9.90 | 34.6 | 97.5 | | 0.10 | 26.7 |
|  | 90 | ‘frem’ | 48.1 | 27.2 | 43.0 | 58.1 | 13.7 | 34.6 | 68.8 | | 2.10 | 26.8 |
|  |  | ‘scm’ | 75.8 | 18.6 | 41.0 | 86.7 | 9.60 | 34.5 | 97.5 | | 0.10 | 26.9 |
| **100** | 0 | ‘frem’ | 89.9 | 7.60 | 29.8 | 96.1 | 2.90 | 25.9 | 100 | | 0.13 | 18.9 |
|  |  | ‘scm’ | 92.5 | 5.10 | 30.1 | 97.7 | 1.00 | 25.9 | 100 | | -0.7 | 18.9 |
|  | 50 | ‘frem’ | 85.4 | 8.4 | 29.9 | 94.0 | 3.40 | 25.8 | 98.7 | | 0.05 | 19.3 |
|  |  | ‘scm’ | 92.5 | 5.10 | 29.8 | 97.7 | 1.10 | 26.0 | 99.8 | | -1.0 | 19.6 |
|  | 90 | ‘frem’ | 67.6 | 7.50 | 30.0 | 76.9 | 3.00 | 25.8 | 81.0 | | -0.5 | 20.1 |
|  |  | ‘scm’ | 92.9 | 4.80 | 30.0 | 98.1 | 0.80 | 26.2 | 99.9 | | -1.1 | 19.8 |


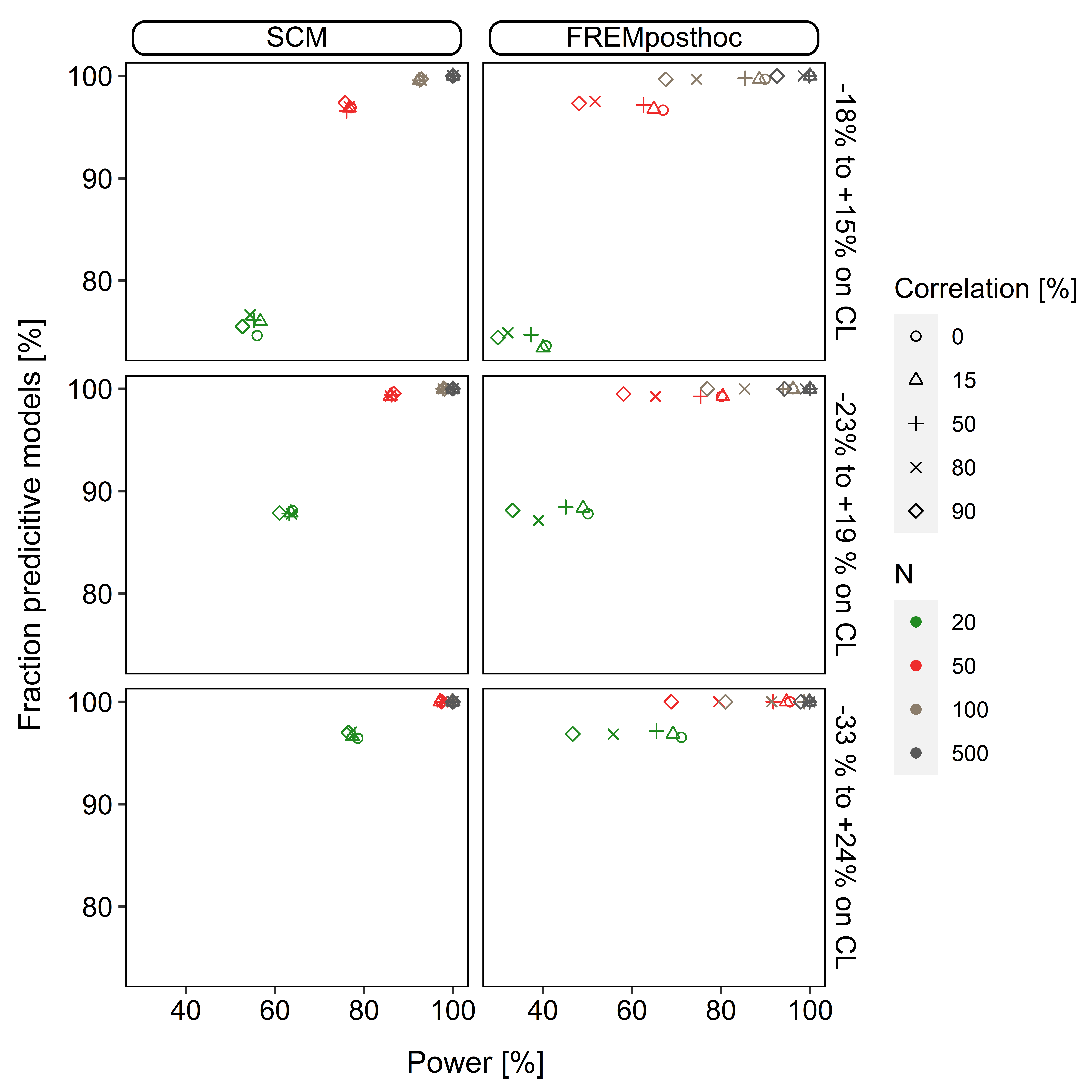


Figure S3 - 4 Fraction of models with high predictive performance for scm and final ‘frem_posthoc_’ models with significant true covariate relationships in scenario 2. Estimated coefficients between zero to two times the true value were assumed to improve the predictive performance.


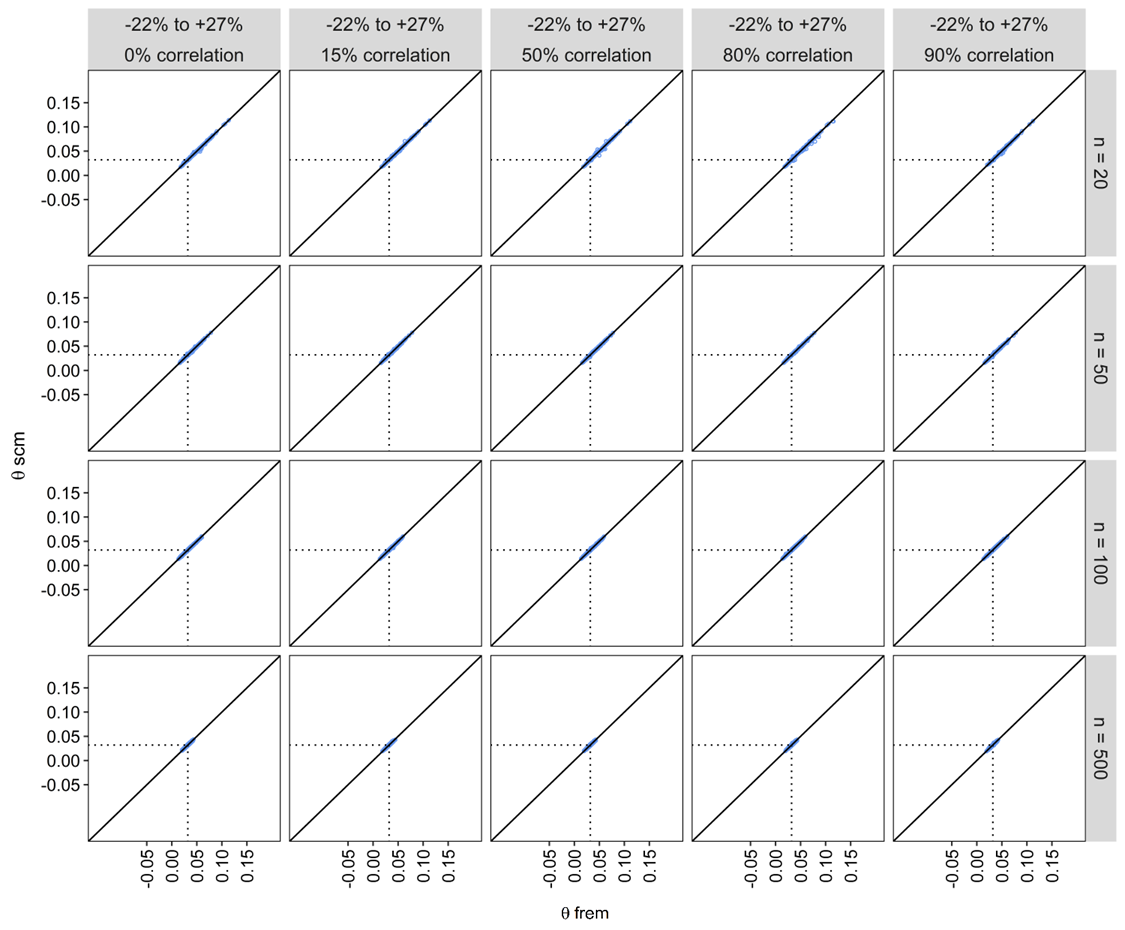


Figure S3 - 5 Estimated ‘scm’ vs. ‘frem_posthoc_’ coefficients per study scenario (θ = 0.032). Dotted line represents the true coefficient value.

**Results for the Scenario 3**

Scenario 3 compared all estimated ‘frem’ cov_true_ coefficients without a posthoc selection against those of the final ‘scm’ model obtained after forward inclusion (p < 0.05) and backward elimination (p < 0.01). In sum all ‘frem’ coefficients are unbiased (-1.4 – 3.7 %) compared to the ‘scm’ where a selection is default. Even if unbiased, the estimated ‘frem’ coefficients are highly imprecise, especially in datasets n < 100. Nevertheless ‘frem’ provides cov_true_ coefficients with a higher precision in small n datasets. Rrmse was reduced from 48 % to 27 % from weakest to strongest covariate effect scenario (n = 50) and from 80 % to 8 % with increasing dataset size (n = 20 – 500). In sum final ‘frem’ model provide a high faction of predictive models (> 87 %), mostly impacted by covariate effect magnitude as shown in Figure S3 - 6.


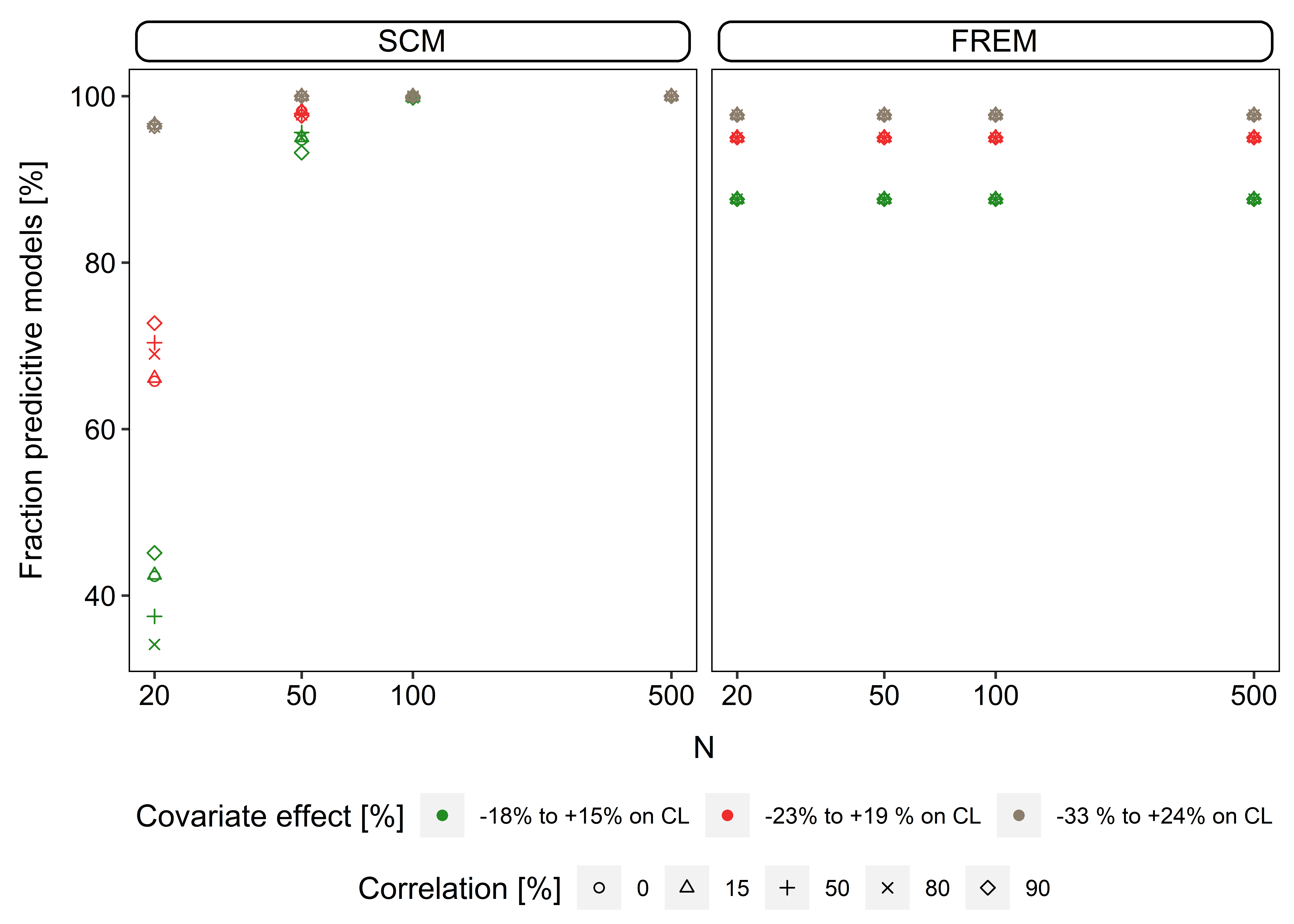


Figure S3 - 6 Fraction of models with high predictive performance for final scm and ‘frem’ models in scenario 3. Estimated coefficients between zero to two times the true value were assumed to improve the predictive performance.
